# Molecular Characterisation of Equine Herpesvirus 1 Isolates from Cases of Abortion, Respiratory and Neurological Disease in Ireland between 1990 and 2017

**DOI:** 10.3390/pathogens8010007

**Published:** 2019-01-15

**Authors:** Marie Garvey, Rachel Lyons, Ralph D Hector, Cathal Walsh, Sean Arkins, Ann Cullinane

**Affiliations:** 1 Virology Unit, The Irish Equine Centre, Johnstown, Naas, Co. Kildare W91 RH93, Ireland; MGarvey@irishequinecentre.ie (M.G.); RLyons@irishequinecentre.ie (R.L.); 2 Centre for Discovery Brain Sciences, University of Edinburgh, Edinburgh EH8 9XD, UK; ralph.hector@ed.ac.uk; 3 Department of Mathematics and Statistics, University of Limerick, Limerick V94 T9PX, Ireland; Cathal.Walsh@ul.ie; 4 Department of Biological Sciences, University of Limerick, Limerick V94 T9PX, Ireland; Sean.Arkins@ul.ie

**Keywords:** EHV-1, multi-locus typing, ORF68, neuropathogenic marker

## Abstract

Multiple locus typing based on sequencing heterologous regions in 26 open reading frames (ORFs) of equine herpesvirus 1 (EHV-1) strains Ab4 and V592 was used to characterise 272 EHV-1 isolates from 238 outbreaks of abortion, respiratory or neurological disease over a 28-year period. The analysis grouped the 272 viruses into at least 10 of the 13 unique long region (U_L_) clades previously recognised. Viruses from the same outbreak had identical multi-locus profiles. Sequencing of the ORF68 region of EHV-1 isolates from 222 outbreaks established a divergence into seven groups and network analysis demonstrated that Irish genotypes were not geographically restricted but clustered with viruses from all over the world. Multi-locus analysis proved a more comprehensive method of strain typing than ORF68 sequencing. It was demonstrated that when interpreted in combination with epidemiological data, this type of analysis has a potential role in tracking virus between premises and therefore in the implementation of targeted control measures. Viruses from 31 of 238 outbreaks analysed had the proposed ORF30 G2254/D752 neuropathogenic marker. There was a statistically significant association between viruses of the G2254/D752 genotype and both neurological disease and hypervirulence as defined by outbreaks involving multiple abortion or neurological cases. The association of neurological disease in those with the G2254/D752 genotype was estimated as 27 times greater than in those with the A2254/N752 genotype.

## 1. Introduction

Equid alphaherpesvirus 1 (EHV-1) commonly known as equine herpesvirus 1, is the most economically and clinically significant equine herpesvirus [1]. EHV-1 belongs to the *Alphaherpesvirinae* subfamily, genus *Varicellovirus* [2]. The virus has a global distribution in horse populations, causing several clinical syndromes including mild to severe respiratory disease, abortion, neonatal foal death, chorioretinitis, and neurological disorders often referred to as equine herpesvirus myeloencephalopathy (EHM) [3,4,5,6]. The neurological form of the disease is less common than abortion or respiratory disease but can result in fatalities [6]. Primary infection occurs via the respiratory epithelium by close contact with infectious animals and inhalation of virus, or in rare cases contaminated feed and water [7,8]. Infection is followed by a cell associated viraemia that is central to the virus spreading to secondary sites of infection [9,10]. EHM results from the inflammatory response to infection of the vascular endothelium of arteries in the central nervous system (CNS), leading to local haemorrhage, thrombosis, and ischaemia [11]. The occurrence of an increase in devastating outbreaks of EHM throughout North America and Europe in 2006 and 2007 led to its classification as a potentially emerging disease by the U.S. Department of Agriculture’s Animal and Health Inspection Service [12]. EHV-1 is a World Organisation for Animal Health (OIE) listed disease which must be notified to OIE so that appropriate action can be taken to ensure safe international trade in horses [13]. 

The EHV-1 genome consists of a linear molecule of double stranded DNA, approximately 150 kbp in length [14]. The genome is composed of a unique long (U_L_) region and a unique short (U_S_) region. These genomic segments are flanked by inverted repetitive elements called terminal (TR_L_ and TR_S_) and internal (IR_L_ and IR_S_) repeats. Traditionally, the genome is separated into 80 open reading frames that encode 76 distinct proteins and four repeated ones. To date, 28 complete EHV-1 genomes isolated from the domestic horse are available in GenBank. Recently the complete genome sequencing of the attenuated Kentucky A strain (KyA) a candidate vaccine strain, revealed two additional open reading frames (ORFs) in the internal repeat region (IR2 and IR3; ORFs 77 and 78) that are conserved in the virulent RacL11 and Ab4 strains [15].

Experimental infections demonstrated that different strains of EHV-1 vary in their abortigenic potential and in their neuropathogenicity [16,17,18,19]. EHV-1 strain Ab4 isolated from a quadriplegic gelding was associated with severe clinical disease including neurological disease and frequent abortion [3]. In contrast, strain V592, isolated from a foetus during a large abortion storm [20], appears to be less virulent on experimental infection, resulting in low levels of viraemia, few cases of abortion, and no neurological disease [16,18]. 

The availability of the genome sequences of Ab4 [14] and V592 [21] encouraged efforts to define the determinants of neuropathogenicity. Forty-two amino acid residue differences across 31 ORFs were identified and a single nucleotide polymorphism (SNP) within the polymerase gene (causing a substitution of asparagine (N) to aspartic acid (D) at amino acid position 752 of ORF30) was found to be strongly associated with the occurrence of EHV-1 neurological disease [21]. Subsequently experimental infection with recombinant viruses with differing polymerase sequences supported the link between the G2254/D752 genotype and neuropathogenicity [22,23]. However other international studies of field isolates yielded conflicting results and indicated that this association was not definitive [24,25,26,27].

Nugent et al. (2006) [21] also proposed that a short region spanning approximately 600 bp of ORF68, which encodes virion protein U_S_2 [28], was a primary strain marker for classifying EHV-1 field isolates into six groups [21]. However, there have been conflicting conclusions regarding the usefulness of this approach for molecular tracking of EHV-1 in other countries suggesting that it may not be an effective substitute for more detailed sequence analysis [24,29,30,31,32,33].

A recent study which included the next generation sequencing of 78 EHV-1 strains (the majority of which originated in the United Kingdom (UK)) and phylogenetic analysis with 26 EHV-1 genome sequences from the United States and Australia [34] suggested that EHV-1 has diverged into 13 distinct U_L_ clades [35]. The study concluded that abortion isolates grouped into nine and neurological isolates, most of which had the G2254/D752 substitution, grouped into five of the 13 clades.

The aim of this study was to analyse and classify EHV-1 strains collected from outbreaks of non-neurological (respiratory disease, abortion and neonatal foal death) and neurological disease in the Irish horse population over three decades. Three isolates from two severe abortion outbreaks in Italy were also included. EHV-1 isolates from 238 outbreaks were characterised based on multi-locus profiling and single representatives from 222 of the 238 outbreaks were further characterised by ORF68 sequencing. Isolates from non-neurological and neurological disease outbreaks in addition to sporadic cases versus multiple case outbreaks were also examined for the presence of the putative neurological marker.

## 2. Results

### 2.1. Multi-Locus Analysis

**Figure 1:**
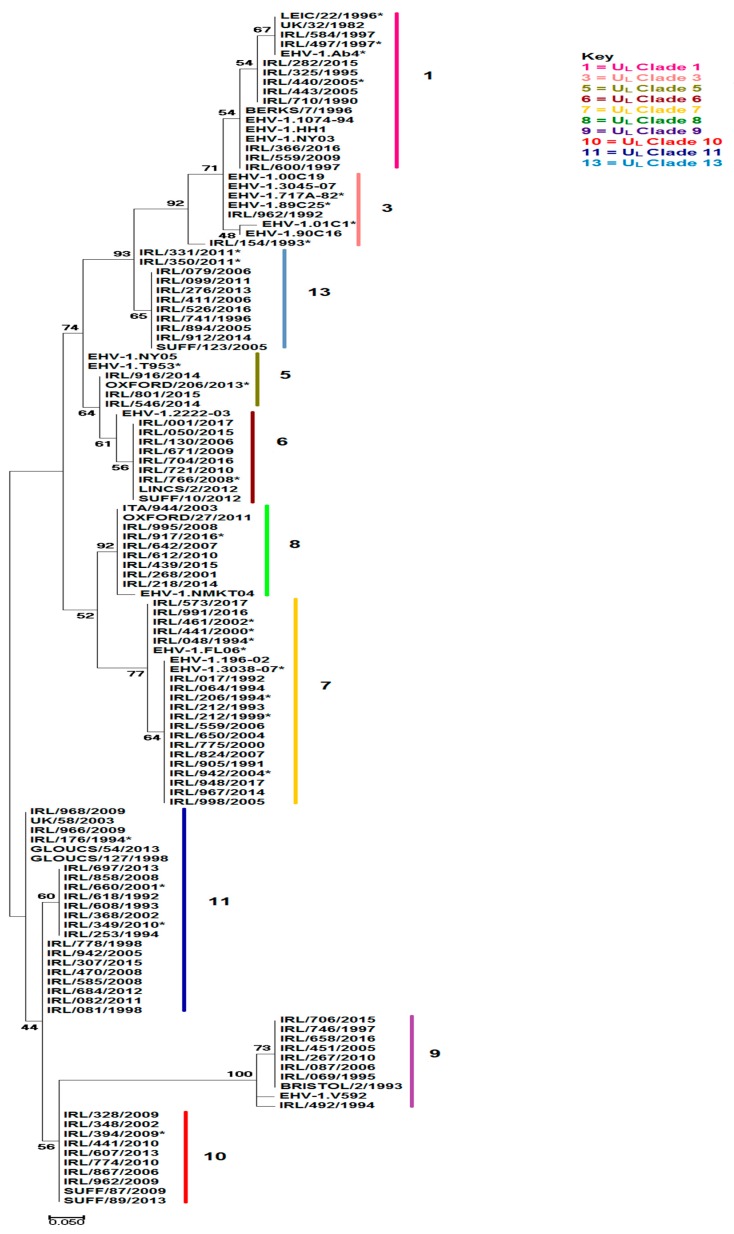
Maximum likelihood phylogenetic tree of 126 representative amino acid sequences based on the Jones Taylor Thornton (JTT) matrix-based model. The sequences were constructed by concatenation of 38 amino acids, 31 non-synonymous substitutions between Ab4 and V592, and seven additional mutations identified in other EHV-1 strains. The tree is based on alignment of the artificial peptide derived by multi-locus sequence typing of 94 EHV-1 isolates sequenced in this study and 32 EHV-1 strains obtained from GenBank with known U_L_ clade grouping [35]. EHV-1 U_L_ clades are indicated by coloured continuous bars and are numbered according to the key. Details of the sequences are in Supplementary Tables S1 and S2. The scale bar represents the number of substitutions per site. Bootstrap values after 100 replications are indicated at major nodes. Asterisks denote neurological isolates.

Phylogenetic analysis (see tree in Figure 1 and alignment in Supplementary Figure S1) was performed using an artificial peptide consisting of concatenated amino acids of U_L_ and U_S_ based on 31 non-synonymous substitutions between Ab4 and V592 and seven additional mutations identified by analysis of viruses characterised in this study and published sequences [34,35]. The analysis grouped the 272 viruses characterised into 10 of the 13 U_L_ clades identified by Bryant et al. (2018) [35]. This approach did not distinguish the single clade 2 representative NY03, which grouped with clade 1 viruses or the single clade 12 representative Suffolk/91/94, which grouped with the clade 10 viruses. Similar to the UK, the majority of Irish isolates (118 isolates from 106 outbreaks from 1991 to 2017) clustered in clade 7 with viruses from the United States and Australia. No Irish viruses belonged to clade 4, which contains the single representative strain RACL11.

One hundred and eleven viruses characterised in this study clustered in clades previously restricted to UK isolates (see Table 1). Isolates from 14 outbreaks (1996 to 2016) clustered in clade 13 which previously had a single representative strain (Suff/123/2005). Isolates from 12 Irish outbreaks (1990 to 2016) and an Italian isolate (2003) clustered with the UK clade 8 viruses, including NMKT04. Other viruses clustered in clades with a wider global distribution, for example isolates from ten outbreaks (eight in Ireland, two in Italy) from 1990 to 2016 clustered with Ab4 and other clade 1 viruses from the UK, Australia, Japan, and Hong Kong. Isolates from 21 outbreaks (2005 to 2017) clustered with clade 6 viruses from the UK, United States, New Zealand, and Australia. Two isolates clustered in clade 3, which was previously restricted to Japanese and Australian isolates.

The number of viruses characterised, and the clades identified for each of the 28 years in the study are summarised in Table 2. Co-circulation of clades was observed but some clades were only identified sporadically. Clade 7 viruses predominated and were identified almost every year. Clades 1, 8, 9, 11 and 13 circulated in all three decades included in the study. In contrast, clade 3 and 5 viruses were confined to 1992/93, and 2014/15 respectively. The clade 3 viruses were isolated on two separate premises in the same county. Similarly, the clade 5 viruses were isolated within a 100-km radius. This suggests that viruses of clades 3 and 5 may have a restricted distribution in Ireland and therefore be easier to trace than viruses of more ubiquitous clades. There was no evidence of circulation of clade 10 and clade 6 strains prior to 2002 and 2005, respectively, but both persisted and were identified up to 2017.

There were 29 premises from which more than one isolate was characterised by multi-locus typing (see Supplementary Table S3). On two premises serial samples collected from the same horse had identical multi-locus profiles. On premises 114 a mare aborted after recovering from neurological disease and the clade 6 viruses isolated from her (IRL/766/2008) and her aborted foetus 10 weeks (IRL/155/2008) later had identical profiles. Similarly, on premises 117, viruses isolated on three separate occasions from a foal born with EHV-1 infection and treated with ganciclovir had identical profiles. Furthermore, the profiles of viruses isolated from different horses during the same outbreak on 15 premises were also identical. However, of 14 premises where outbreaks occurred in different years the viruses isolated were only identical in four. 

The potential usefulness of multi-locus analysis to corroborate or disprove a hypothesis based on epidemiological data was demonstrated in selected outbreaks (see Supplementary Table S1). The hypothesis that multiple cases of neurological disease identified on two different sport horse premises (141 and 142) were linked by horse movement was supported by the identical multi-locus profiles of the viruses isolated (IRL/350/2011 and IRL/331/2011). Similarly, IRL/394/2009 was isolated on premises 121 from a mare with neurological disease that had returned home from a public stud farm, premises 122. Subsequent investigation indicated that IRL/394/2009 had an identical multi-locus profile to virus IRL/626/2009, which was circulating sub-clinically on premises 122. Multi-locus typing also corroborated the hypothesis that in one case of neurological disease the source of virus was reactivation of a latent infection. The yearling of a mare that was present on premises 1 during a severe neurological outbreak travelled to premises 22 the following year to be castrated. A pony that shared a field with the yearling post castration, presented with neurological signs approximately two weeks later. The virus isolated from the in-coordinate pony had the same genetic profile as the viruses associated with EHV-1 outbreaks on premises 1, supporting the hypothesis that reactivated virus from the yearling was the source of infection. In contrast, multi-locus typing provided evidence to disprove the hypothesis that horse movement from premises 15 where IRL/176/1994 was isolated prior to diagnosis of EHV-1 abortion and multiple cases of neurological disease, was the source of virus to a public stud (premises 16) where a single mare developed neurological disease. However, the virus isolated IRL/206/1994 (clade 7) from the single case on the stud farm was readily distinguishable from IRL/176/1994 (clade 11), indicating that premises 15 was highly unlikely to be the source.

**Figure 2:**
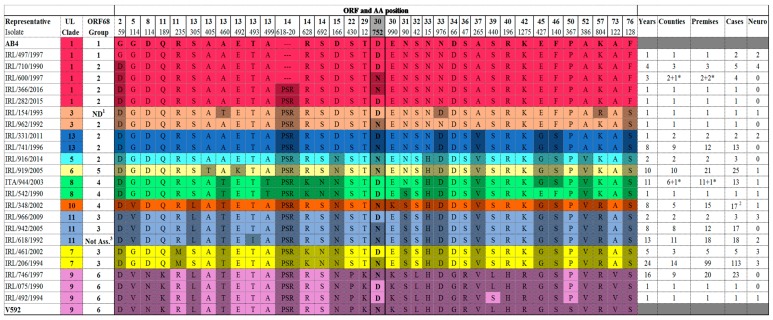
Multi-locus sequence analysis of representative Irish EHV-1 isolates using 38 amino acid differences in 26 open reading frames (ORFs). Amino acid differences (*n* = 38, including triplet in ORF14) between EHV-1 strains Ab4 and V592 and representative Irish isolates across 26 ORFs. Amino acid positions are numbered according to V592. Colours are used to highlight different U_L_ clades [35]. EHV-1 representatives from 10 clades are shown. Shading is used to highlight amino acid difference at that site. The putative neurological marker at ORF30 variable site N752/D752 is highlighted in grey. The number of isolates with the same genotype for each clade is summarised by years, counties, premises, and cases of EHV-1 examined. Neuro indicates number of isolates from cases of neurological disease. ^1^ ND: indicates not determined. ^2^ Includes repeated samples (*n* = 3) from the same case. ^3^ Indicates ORF68 not assigned group. (^---^) represents gap in sequence. * represents an Italian isolate.

For routine molecular epidemiological investigation in a diagnostic laboratory alignment of representative EHV-1 isolates from all clades (see Figure 2) identified that a limited number of non-synonymous sites in six ORFs could be targeted to distinguish the clades identified in this study. Clade 7, the most prominent clade circulating, is readily identified using ORF11 (R235M). The same ORF can be used to identify clade 9 viruses. ORF13 identifies clades 6 and 8 using non-synonymous changes at A405T and A499T, respectively. Sequencing of ORF30 in addition to ORFs 11 and 13 identifies clades 10 and 11. Inclusion of ORFs 37, 52 and 76 identifies the remaining clades 1, 3, 5, and 13. This approach has the potential to be used globally as the clades are not specific to Irish isolates but include viruses from Asia, Australia, North America, and Europe. Furthermore, the assay could be modified in the future employing a multiplex PCR to amplify several target fragments simultaneously prior to sequencing. This would be less labour-intensive and more cost-effective. 

Neurological disease was associated with viruses from nine of the ten clades identified. Outbreaks with multiple cases of abortion were also associated with viruses from nine clades but outbreaks with multiple neurological cases were restricted to viruses from six clades. A significant association between clade and neurological disease was found (*p*-value = 0.02) driven by high expression in clades 1 and 3. Furthermore, the statistical association between clade and hypervirulence was found (*p*-value = 0.027) driven by high expression in clades 1 and 8. 

Thirty-five viruses from 31 outbreaks i.e., 13% of the outbreaks included in the study, had the G2254/D752 change in polymerase gene associated with neuropathogenicity [21]. These viruses clustered in several different clades; eight from six outbreaks were clade 1, one was clade 3, five were clade 7, 14 from 13 outbreaks were clade 8, two were clade 9, three from two outbreaks were clade 11, and two were clade 13 (see Table 1). A statistical association between clade and the G2254/D752 genotype was found (*p*-value < 0.001) driven by high expression in clades 1 and 8. Eighteen of the 35 G2254/D752 viruses (51.4%) were isolated from cases of neurological disease. The remainder were from cases of abortion or neonatal foal death. Six were from five outbreaks of multiple abortion. In this study seven viruses characterised from cases of neurological disease lacked the neuropathogenic marker, i.e., had the A2254/N752 genotype. However, to the best of our knowledge all were single cases. The association between the G2254/D752 genotype and neurological disease was found to be highly significant (*p*-value <0.001). The odds of developing neurological disease after viral infection for those with the G2254/D752 genotype was estimated as 27 times greater than for those with the A2254/N752 genotype (odds ratio (OR) = 26.9; 95% confidence interval (CI): 10–75). Multiple outbreaks of neurological disease were 38 times more likely than single outbreaks if associated with the neuropathogenic marker (OR = 38.3; 95% CI: 2–820). Furthermore, there was a statistically significant association between the G2254/D752 marker and hypervirulent disease expression as defined by outbreaks involving multiple abortion or neurological cases (*p*-value <0.001). The odds ratio for the G2254/D752 genotype and hypervirulence was determined as almost five times greater than with an A2254/N752 strain (OR = 4.8; 95% CI: 2.2–10.5).

### 2.2. ORF68

**Figure 3:**
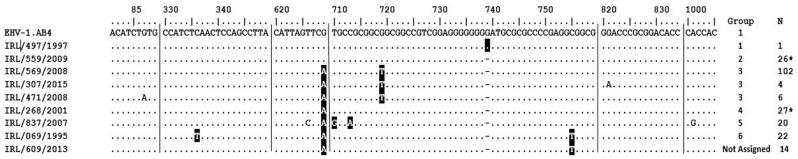
Regions of sequence variation in ORF68 for representative Irish isolates. Analysis of 222 EHV-1 isolates showed they belong to seven of the groups previously described [21]: groups 1–6 and one of the two unassigned groups. *N* indicates the number of isolates characterised with a particular sequence in the group. Dots indicate sequence identity, while group-specific single nucleotide polymorphisms (SNPs) are highlighted. Vertical dashed lines represent breaks in continuous sequence where no changes occurred. The numbers above the alignment indicate the nucleotide positions according to the ORF68 sequence of strain Ab4 (group 1), which contains 8 G residues in the homopolymeric tract (nucleotides 732–739). Symbol (-) denotes nucleotide deletion and (*) denotes includes one Italian strain.

A ~1185 bp nucleotide sequence of ORF68 for 222 isolates representative of 222 EHV-1 outbreaks over the 28-year period was determined to assess the relationship between ORF68 groups and clades identified by multi-locus analysis. Sequence analysis of this polymorphic region of ORF68 identified isolates in each of Nugent’s six groups and one of her two unassigned groups (see alignment Figure 3). ORF68 representative sequences have been deposited in GenBank with accession numbers MH976701-MH976709. Of the 222 isolates analysed using this grouping system, only one Irish isolate belongs to group 1. Similar to Ab4, IRL/497/1997 encodes 8 G’s in the homopolymeric tract, giving rise to a 418 amino acid (aa) long U_S_2 protein compared with 303aa for EHV-1 isolates in other groups. Twenty-six isolates (11.7%) belong to group 2. The majority of isolates 112 (50.45%) belong to group 3 and have the characteristic SNP T719. However, two previously unidentified nucleotide changes at SNP A87 and SNP A821 in group 3 viruses may represent two new sub-groups. Twenty-seven (12.16%) and 20 isolates (9%) belong to groups 4 and 5, respectively. All the group 5 isolates identified had the additional SNP C626 in addition to the group 5 characteristic SNPs G710 and A713. Twenty-two (9.9%) isolates belong to the V592-like group 6. A further 14 isolates (6.3%) which contained A629 and T755 SNPs were categorised as belonging to Nugent’s unassigned group.

**Figure 4:**
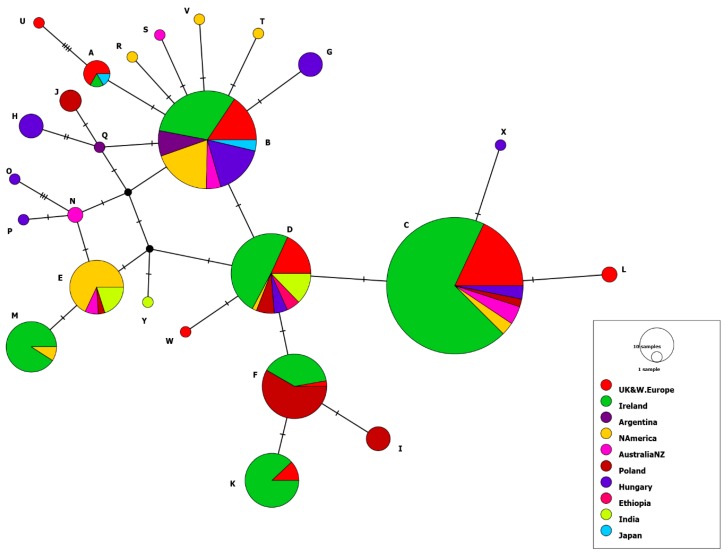
Median joining network of global EHV-1 sequences based on a 464 bp alignment of ORF68 sequences. The network includes 221 Irish isolates, 1 Italian isolate and 219 EHV-1 ORF68 international sequences retrieved from GenBank (see Supplementary Table S4 for sequence information). Nodes, labelled with capital letters (A to Y), represent the same ORF68 sequence and are coloured based on the geographical origin of the sample. The area of each circle is in proportion to the frequency of isolates sharing the same genotype. Hatch marks (|) represent segregating sites. Abbreviations: UK&W.Europe: United Kingdom and Western Europe; NAmer: North America; AustraliaNZ: Australia and New Zealand.

Network analysis (Figure 4) demonstrated that there was no correlation between the sequence of the polymorphic region of ORF68 and the location of the country where the virus was isolated, as the majority of Irish isolates clustered with viruses from all over the world. The single Irish group 1 isolate, IRL/497/1997, clusters with four UK isolates (one of which is Ab4) and one Japanese respiratory isolate in node A. Twenty-five group 2 Irish isolates cluster in node B with viruses from Europe, Japan, North America, Argentina, and Australia. One hundred and twelve group 3 isolates share the largest node C with isolates from Europe, North America, and Australia. A further 27 group 4 isolates cluster with European, North American, Ethiopian, and Indian strains in node D. All 20 Irish group 5 isolates share node M with North American strains. Node K contains only three strains from the UK, including reference strain V592 in addition to 22 Irish isolates. Finally, 14 Irish strains belonging to Nugent’s unassigned group shared node F with a single UK strain and 21 Polish strains only. The single Italian isolate analysed belonged to node B (group 2).

There was correlation of ORF68 groups and clades identified by multi-locus analysis. The single ORF68 group 1 isolate IRL/497/1997, which is similar to Ab4, has G8 in the homopolymeric tract, clustered with clade 1 viruses. Within ORF68 group 2 there were representatives from clades 1, 3, 5, and 13. Group 3 contained all the clade 7 viruses and 10 of the 24 clade 11 viruses characterised. Six clade 11 viruses had an additional SNP A87 which was absent in clade 7 viruses. Group 4 contained all clade 8 and clade 10 viruses identified. Groups 5 and 6 contained clade 6 and 9 viruses, respectively. The unassigned group described by Nugent et al. (2006) [21] with SNPs A629 and T755 was composed of 14 clade 11 viruses.

## 3. Discussion

The present study is the first to document the molecular characterisation of EHV-1 clinical isolates in Ireland over a 28-year period. Two hundred and sixty-nine viruses detected in Ireland and a further three viruses isolated in Italy were included in the multi-locus analysis. Our investigation established that genetic characterisation has the potential to be a useful aid in the management of EHV-1 outbreaks, based on identification of the G2254/D752 polymerase genotype, the U_L_ clade assignation, and to a lesser extent, ORF68 sequencing.

Multi-locus typing of EHV-1 was initiated by Nugent et al. (2006) [21] and extended in this study to allow comparison to recently proposed U_L_ clades [35]. In the original multi-locus study, analysis of a panel of twenty-five isolates (12 neurological and 13 non-neurological) using the amino acid differences between EHV-1 reference strains Ab4 and V592 led to the proposal that ORF68 analysis could be used for distinguishing isolates without having to type multiple loci [21]. Consequently, multi-locus analysis has not been widely used by other investigators who have concentrated on ORF68 sequencing. In this study, multi-locus typing of 272 EHV-1 isolates established a correlation to 11 of the 13 EHV-1 clades proposed by the recent UK genotyping study [35]. The 272 isolates characterised clustered in 10 of the 11 clades. Our results concur with those of Bryant et al. (2018) [35] in demonstrating that clade 7 viruses predominate, and that simultaneous co-circulation of clades occurs in Ireland. In the next generation sequencing (NGS) study by Bryant et al. (2018) [35] network analysis suggested that recombination had occurred between EHV-1 strains. This has also been observed in herpes simplex virus 1 (HSV-1) strains [36]. Furthermore inter-species recombination has been detected in field samples between EHV-1 and EHV-4 [34,37], EHV-1 and EHV-9 [38], and between EHV-1 and EHV-8 [35]. Thus, although the genotyping method based on the targeted multi-locus approach used in this study and also recently developed for VZV [39] may be more practical than NGS for surveillance purposes, it has the limitation that it does not allow the detection of possible recombination crossovers in the unanalysed parts of the genome [40]. Whole genome sequencing (WGS) of viruses is increasingly important in clinical settings but is not yet routinely used in the majority of veterinary diagnostic laboratories. As sequencing costs continue to decrease, specialised bioinformatic resources become more accessible and methods are standardised, WGS using NGS methods is likely to be more widely applied in veterinary medicine providing molecular epidemiology studies greater accuracy [41]. Meanwhile however, targeted PCR amplification and Sanger sequencing offer a rapid and robust alternative for the detection of virus variants. This study indicated that analysis of a SNP identifies viruses of the most common EHV-1 clade in the UK and Ireland, and that only six ORFs need to be targeted to discriminate between ten clades. 

In this study the multi-locus analysis proved very useful to support or negate the epidemiological data in the tracking of virus between selected premises. Viruses from the same outbreak had identical profiles whereas viruses identified on the same premises in different years were rarely identical suggesting reintroduction rather than reactivation or persistent circulation. In three outbreaks on premises with an epidemiological link the viruses had identical profiles. This included a case of suspected reactivation of virus from an outbreak in the previous year in a different province. In addition to providing support for epidemiological links the multi-locus analysis also provided conflicting evidence that a premise with multiple neurological cases was the source of virus linked to a single case on another premises. 

In the future it is envisaged that molecular typing will become routine in our laboratory and as several of the EHV-1 clades are not geographically restricted, this approach can be used in other countries. Molecular evidence corroborating equestrian events or specific premises as the source of virus will assist in the implementation of targeted movement restrictions, quarantine and other control measures. The results of such analysis are not proof of a causal link but add strength and depth to a clinical advisory service. If for example, viruses of the same clade are isolated from cases on different premises linked directly or indirectly to return of horses from a training centre or public stud farm it becomes incumbent on the owner of that centre or farm to communicate a possible risk to clients. In terms of clinical management an informed decision may be taken to quarantine horses on the public premises until there is no further evidence of circulating virus or clients may implement extra biosecurity measures in relation to transport, isolation and monitoring of horses returning from that centre. Demonstration that repeated outbreaks of EHV-1 on individual premises are due to viruses of different clades can also contribute to clinical management. Owners of premises that suffer repeated incidences of EHV-1 associated disease frequently focus on reactivation of latent virus and identification and removal of a “carrier”. Molecular evidence to the contrary facilitates the introduction of improved management practices with respect to vaccination, separation of broodmares from younger stock and sport horses and temporary isolation of visiting mares. 

Since Nugent et al. (2006) [21] put forward the hypothesis that variants with the G2254/D752 substitution in the DNA polymerase have increased likelihood of association with neuropathogenicity, there has been international focus on the characterisation of the genotypes of EHV-1 isolates and allelic discrimination assays have been widely used to distinguish between neuropathogenic and non-neuropathogenic strains [42,43]. Many studies concentrate on the retrospective investigation of archived viruses and the prevalence data generated must be interpreted with caution due to sampling and storage bias. In this study, 35 of 272 viruses from 238 outbreaks had the G2254/D752 genotype suggesting a prevalence of 12.9% in Ireland. However, this prevalence may reflect sample bias for the isolates chosen for characterisation, prior to the introduction of routine genotyping in 2005. Thus, the true prevalence is likely to be nearer 9% calculated from the years 2005–2017. The findings indicate that the vast majority of Irish viruses have the A2254/N752 genotype. It has been demonstrated in some studies that horses infected with a G2254/D752 variant such as Ab4 show higher levels of virus shedding than horses infected with the A2254/N752 variant such as V592 [16,19,44,45,46]. Subsequently it was suggested that this may indicate a selective advantage of the G2254/D752 strains which could favour an increase in prevalence such as that reported in the United States from 3.3% in the 1960s to 19.4% since the year 2000 [26,47,48]. In recent years there has been an increase in the number of severe EHM outbreaks reported in other countries including France which subscribes to a Tripartite Agreement for the free movement of horses without health checks between Ireland, France, and the UK [49,50]. However, neither a parallel increase in the incidence of EHM nor an increase in the detection of the G2254/D752 variant has been observed in Ireland. The abundance of A2254/N752 variants in the majority of field studies globally suggests that the proposed selective advantage of the G2254/D752 variant has not resulted in strain displacement in the wider equine population [25,35,48,49,51,52]. 

Viraemia is essential for the spread of the virus from peripheral blood mononuclear cells (PBMCs) to endothelial cells lining the blood vessels in the CNS or the pregnant uterus [11,53]. Horses experimentally infected with neuropathogenic strains develop a cell-associated viraemia greater in magnitude and longer in duration than with non-neuropathogenic virus strains [16,19,54] and G2254/D752 strains are more successful in the infection of PBMCs and the establishment of viraemia compared to A2254/N752 strains [46,55]. In this study there was a statistically significant association between viruses of the G2254/D752 genotype and hypervirulent disease expression as defined by outbreaks involving multiple abortions or neurological cases. International findings related to the association of EHV-1 genotype with pathogenic phenotypes vary. Nugent et al. (2006) examined 131 EHV-1 isolates from nine countries [21]. Of the 49 neurological isolates examined, 42 (86%) had the G2254/D752 genotype whereas 78/82 (95%) of non-neurological isolates had the A2254/N752 genotype. Following this study several large outbreaks of EHM documented in the literature were associated with the G2254/D752 genotype including outbreaks in Croatia [56], France [49], Germany [57,58], Canada [44] and the first reported outbreak of EHM in New Zealand [59]. However, studies in other countries showed that not all horses with EHM were infected with a strain of the G2254/D752 genotype and that A2254/N752 variants are also associated with neurological disease [24,26,52,60]. Similarly, the G2254/D752 genotype was associated with non-neurological/abortion outbreaks in Europe [25,51,60] and the Americas [48,52]. In this study, the likelihood of neurological disease was 27 times greater when the causal virus was of the G2254/D752 genotype rather than the A2254/N752 genotype. However, the onset of neurological disease cannot be fully attributed to this virus polymorphism and it is suggested that other viral pathogenicity determinants such as glycoprotein D and host factors such as age, gender, immunity and hormonal status may contribute to disease severity [46,61]. More recently Brosnahan et al. (2018) [62] investigated the role of host genetics and identified a SNP in an intron of a platelet-related gene associated with EHM. 

Since 2006, when it was first proposed by Nugent et al. (2006) [21] that the ORF68 polymorphic region was a putative molecular marker for epidemiological studies, this region has been commonly used for genotyping of EHV-1 isolates in different countries: Australia [24], Ethiopia [31], Hungary [30], India [29], Japan [33], and Poland [32]. The study by Nugent et al. (2006) [21] identified six major groups (1–6) and two unassigned groups based on analysis of 106 global isolates and proposed that certain strain groups were geographically restricted. Sequence analysis in this study showed that all Irish isolates segregated into the six groups and one of the two unassigned groups described by Nugent et al. (2006) [21]. The majority of viruses characterised internationally also support this ORF68 grouping system. Cuxson et al. (2014) classified 52 Australian isolates as group 2 or 3 and two as group 5 [24]. Ninety-one Ethiopian isolates were restricted to group 4 [31], eight Indian isolates clustered within groups 4 and 5 [29] and a Japanese isolate was classified as group 2 [33]. However, several of these studies also reported a small number of viruses that could not be classified within the original proposed groups [24,29,33]. Studies in Eastern Europe identified further polymorphism. A study of 38 viruses from cases of abortion in Poland assigned three to group 3, four to group 4, and 22 to one of the unassigned groups [21] but nine were classified in two novel groups [32]. Similarly, in Hungary only 23 of 35 isolates fitted with the originally described groups (groups 2, 3, and 4) and four new groups were proposed [30]. None of the viruses in this study grouped in the novel groups proposed.

The original hypothesis that ORF68 groups are geographically restricted is not supported by the results of our study or those of other investigators. For example, Nugent et al. (2006) [21] found that all group 5 isolates came from outbreaks in North America; however, 9% (20/222) of Irish isolates belong to this group, which has been demonstrated to include viruses from Australia [24] and India [29]. Network analysis of Irish isolates with international strains showed that Irish isolates clustered within 7 nodes with isolates from the several different geographic regions. In agreement with the conclusions from studies in Hungary and Poland this suggests that ORF68 is not a suitable global marker [30,32]. However, this type of strain variation has been demonstrated to be a useful adjunct to epidemiological data when investigating disease outbreaks on multiple premises [56,57,63]. In this study ORF68 genetic analysis of Irish isolates substantiated virus tracking by multiple-locus typing. However, analysis of 222 isolates by both ORF68 sequence and multi-locus typing indicated that although both are useful molecular epidemiological tools multi-locus typing is more accurate. The ORF68 grouping system groups together some viruses from different clades which are readily distinguishable by multi-locus analysis. As more EHV-1 strains are sequenced internationally, additional polymorphisms and new clades are likely to be identified, with the potential to further refine epidemiological investigations in identifying transmission pathways.

In conclusion, this is the first study to explore the genetic diversity of EHV-1 in Ireland, the third largest producer of thoroughbred foals in the world [64]. The contribution of genetic characterisation to our understanding of viral pathogenesis, development of diagnostics, implementation of evidence-based management strategies, and predictions of likely outcome and disease spread is increasing. The data relating to over 250 EHV-1 isolates presented here adds depth to our knowledge of circulating genotypes and illustrates that tracking of virus by genetic analysis when used in combination with epidemiological data gives valuable insights and support for targeted preventive measures. An example of a targeted preventive measure is where on acceptance of data implicating mare sales as the transmission pathway for EHV-1 abortions at geographically disparate locations, a sales company introduces a new condition of sale that all pregnant mares are vaccinated against EHV-1. Consistent with previous studies globally, our results indicated that infection with a strain of the G2254/D752 genotype will not inevitably result in neurological disease. Nevertheless, the strong association with hypervirulence observed in this study suggests that it would be of benefit to veterinarians to be aware that horses in their care are at increased risk of developing EHM or multiple abortions when a virus of this genotype is detected. 

## 4. Materials and Methods 

### 4.1. Viruses

EHV-1 viruses archived at the Virology Unit of the Irish Equine Centre between 1990 and 2017 were retrieved along with the clinical histories available. The clinical samples, which had been stored at −70 °C, included nasal secretions and tissue homogenates from cases of neurological disease, abortion, and neonatal foal deaths. Two hundred and sixty-nine isolates originated from Ireland. Three isolates from two severe abortion outbreaks in Italy were also included. The samples were retrospectively allocated a unique reference number derived from the country, laboratory number and year of collection. A summary of the numbers of isolates analysed in this study is given in Table 3. An overview of all samples included in this study is given in Supplementary Table S1. Two hundred and seventy-two isolates (269 horses) from 238 outbreaks on 220 premises were genetically characterised by multi-locus typing. Representative viruses from 222 of these 238 outbreaks were also characterised by ORF68 sequencing. 

The majority of the viruses were recovered from clinical samples: nasal secretions from respiratory/neurological disease (*n* = 10) and tissues (lungs, liver, spleen, allantochorion, and amniotic cord) from cases of abortion/neonatal foal death (*n* = 237). Twenty-five samples with a low concentration of virus (brain tissue from cases of neurological disease (*n* = 2), nasal swabs (*n* = 14) and multiple tissues (*n* = 9)) were amplified by a single passage in cell culture. For culture isolation of these viruses, 25 cm^3^ tissue culture flasks of near confluent rabbit kidney (RK-13) cells were inoculated with 0.5 ml tissue homogenate/nasal fluid. The cells were maintained in 5 ml of maintenance medium (supplemented with 2% foetal calf serum) at 37 °C in an atmosphere of 5% CO_2_ [65]. The monolayer was examined for the presence of cytopathic effect (CPE). Where sequences were compared between DNA samples prepared directly from the sample and tissue culture isolates from the same outbreak, they were found to be identical (*n* = 10).

### 4.2. Extraction of DNA 

DNA was extracted from 200 µL of tissue homogenates/nasal secretions/RK-13 infected cells using the QIAamp DNA Mini kit (Cat No: 51306, Qiagen) according to the manufacturer’s instructions. Alternatively, DNA was extracted from 100 µL of sample by an automated method using the Kingfisher Flex Magnetic Particle Processor instrument (Thermo Scientific) with the LSI MagVet Universal Isolation Kit (Life Technologies) as per the kit manufacturer’s guidelines. 

### 4.3. PCR of multiple loci of EHV-1

The complete genome sequences of Ab4 (AY665713.1) and V592 (AY464052.1) were aligned using ClustalW [66]. Non-synonymous changes were identified between the two genomes in the protein coding regions (as described previously [21]) and primers were designed with similar annealing temperatures to amplify the loci of sequence variation between Ab4 and V592 (see Table 4). Primers were not designed for ORF24 and ORF71 as these contained repeat regions considered to be of limited use for epidemiological studies [21]. Primer sequences are detailed in Supplementary Table S5. The reaction component (50 µL) for amplification of the target sequences consisted of 2.5 U GoTaq Hot Start Polymerase (5 U/µL) (catalogue no. M5001, Promega), 1X GoTaq Flexi Buffer, 2 mM MgCl_2_ solution, 0.2 mM dNTP Mixture (Applied Biosystems), 0.4 of µM each primer, 5 µL template DNA, and nuclease free water (NFW). The cycling conditions were as follows: an initial denaturation at 95 °C for 5 min followed by 40 cycles of denaturation at 95 °C for 30 s, annealing at 57 °C for 45 s, elongation at 72 °C for 2 min, and final extension at 72 °C for 5 min. 

Post-amplification 5 µL of each PCR product was analysed on a 1.2% agarose gel (Sigma) stained with 0.003% Sybersafe (Invitrogen). Reactions were purified using the QIAquick PCR Purification kit (catalogue no. 28106, Qiagen) or the QIAquick Gel Extraction kit (catalogue no. 28706, Qiagen). Purified PCR products were sequenced using Sanger dideoxynucleotide sequencing technology (MRC-University of Glasgow Centre for Virus Research, Glasgow, UK; GATC-Biotech, Cologne, Germany).

### 4.4. Multi-Locus Sequence Analysis

Nucleotide sequences obtained from targeted multi-locus sequence analysis were aligned to individual ORFs of reference strains Ab4 (neuropathogenic, G2254/D752 strain) and V592 (non-neuropathogenic, A2254/N752 strain) using Seqman. Comparative analysis of predicted partial amino acid sequences was carried out for each isolate by using the ClustalW [66] accessory application in BioEdit sequence alignment editor version 7.2.5 [67]. Twenty-eight complete and 78 partial EHV-1 genome sequences which had been included in the study by Bryant et al. (2018) [35] were mined from GenBank [68] (Supplementary Table S2). Nucleotide sequences of individual ORFs were translated using ClustalW implemented in Bioedit. Multiple amino acid sequence alignments were produced for individual ORFs with Ab4 as a reference sequence. Positions of amino acid sequence variation were recorded and tabulated for the sequenced isolates and EHV-1 genome sequences.

Thirty-one non-synonymous substitutions between Ab4 and V592 in 26 ORFs in U_L_ and U_S_ were examined. Amino acid alignments of complete genome sequences and isolates sequenced in this study identified seven additional substitutions to those in Ab4 and V592 in ORFs 11 (R235M), 13 (A405T, E492K, T493I, A499T), and 14 (R628K, S692N) which were also included in the analysis. A concatenated amino acid sequence based on these 38 amino acid differences was constructed for each isolate and the EHV-1 genome sequences. The resulting 38aa artificial peptide sequences (*n* = 321) were aligned using ClustalW (Supplementary Figure S1). Representative sequences (*n* = 126, including 96 isolates sequenced in this study) were aligned in MUSCLE [69] implemented in MEGA7 version 7.0.14 [70]. Phylogenetic analysis of these 126 sequences was inferred by the Maximum Likelihood method based on the Jones Taylor Thornton (JTT) matrix-based model [71] with bootstrap values determined over 100 iterations. The topology of the tree was examined for U_L_ clade resolution based on the study of Bryant et al. (2018) [35]. 

### 4.5. PCR of EHV-1 ORF68 

PCR primers were designed to amplify ORF68 (US_2_) (detailed in Table 5) based on EHV-1 reference sequences Ab4 (GenBank accession AY665713.1) and V592 (GenBank accession AY464052.1) using the online application Primer3 [72]. Amplification was performed using the G-Storm (Gene Technologies) with the PCRx Enhancer System (catalogue no. 11495-017, Invitrogen) which is specific for the amplification of problematic and/or GC-rich templates. The reaction component consisted of 1X PCRx Amplification Buffer, 1.5 mM MgSO4, 2X PCRx Enhancer Solution, 0.4 µM each primer, 5 U of Taq DNA Polymerase (5 U/µL, Invitrogen), 0.2 mM dNTP Mixture (Applied Biosystems), 10 µL of template DNA, and nuclease free water to a final volume of 50 µL. Initial denaturation was carried out at 95 °C for 5 min, followed by amplification with 40 cycles of 95 °C for 30 s, 50 °C for 1 min, 72 °C for 2 min, and a final elongation at 72 °C for 10 min.

### 4.6. ORF68 Sequence Analysis

The four overlapping sequence regions of the 1313bp ORF68 amplicon were assembled for each isolate using Seqman Version 5.01 (DNASTAR). Nucleotide sequence alignments were performed using the ClustalW [66] application in BioEdit [67]. Sequences were aligned using Ab4 strain as a reference to identify variable positions and perform grouping of the isolates as per Nugent et al. (2006) [21]. Isolates from 222 of the 238 outbreaks, defined as an occurrence of one or more cases in an epidemiological unit, were included in the analysis. Incomplete ORF68 sequence data was obtained for 16 of the 238 outbreaks which necessitated exclusion from the analysis. ORF68 sequence data for a further 219 EHV-1 isolates were retrieved from GenBank (sequence information can be found in Supplementary Table S4). The ORF68 alignment of 464 bp in length was converted to nexus file format using Seqret in EMBOSS [73]. An international median-joining haplotype network of EHV-1 ORF68 sequences, colour-coded by geographic location, was constructed in PopART version 1.7 [74] as described previously for Polish EHV-1 strains [32]. 

### 4.7. Statistical Methods 

A chi-squared test for association and proportion was used to test the null hypothesis that there was no difference in the relative proportions of the G2254/D752 genotype between: isolates originating from neurological and non-neurological outbreaks, isolates originating from single cases of neurological disease and outbreaks with multiple neurological cases, and isolates originating from hypervirulent disease expression defined as multiple abortions or multiple cases of neurological disease, and those from sporadic cases. The same statistical test was used to investigate the statistical significance of: the G2254/D752 genotype, hypervirulent disease expression, and neurological disease, across the clades where Irish strains resided. Odds ratios for neurological disease, hypervirulent disease expression, and multiple neurological cases with the putative neurological marker were estimated with a 95% confidence interval. The data was summarised in 2 × 2 contingency tables and analysis was conducted in the R statistical software package version 3.5.1. The statistical significance was set at α = 0.05.

## Figures and Tables

**Table 1:** Summary of multi-locus sequence typing results of the 272 equine herpesvirus 1 (EHV-1) isolates characterised.

						Abortion		Neurological	
U_L_ Clade ^1^	Isolates	Outbreaks	Premises	ORF30 D752 ^3^	Respiratory	Single ^4^	Multiple ^4^	Single	Multiple
1	13	10	9	8	0	4	2	1	3
3	2	2	2	1	0	1	0	1	0
5	3	2	2	0	0	1	1	0	0
6	25	21	21	0	1	19 *	1	1 *	0
7	118	106	104	5	1	80	19	5	1
8	14	13	13	14	0	7	4	0	2
9	25	22	22	2	0	16	5	0	1
10	19	16	15	0	0	12	3	1	0
11	38	32	32	3	1	23	4	2	2
13	15	14	14	2	0	8	4	0	2
**Total**	**272**	**238**	**220 ^2^**	**35**	**3**	170 *	**43**	11 *	**11**

^1^ Unique long region (U_L_) clade number correlates to those previously described [35]. ^2^ Some premises experienced outbreaks in different years. Outbreaks from a total of 220 premises were investigated. ^3^ Represents the number of isolates with the putative neurological marker G2254/D752 in the polymerase catalytic subunit open reading frame (ORF) 30 [21]. ^4^ Single is defined as one case on a premises. Multiple is defined as more than one case on a premises. * One mare is categorised under single abortion and single neurological disease expression, as isolate IRL/766/2008 was collected when she exhibited neurological signs and isolate IRL/155/2008 was collected from her aborted foetus 11 weeks later.

**Table 2:** The EHV-1 U_L_ clade distribution according to year (1990–2017).

Year	Clade 1	Clade 3	Clade 5	Clade 6	Clade 7	Clade 8	Clade 9	Clade 10	Clade 11	Clade 13	Yearly Total
1990	1					1	1				3
1991					2						2
1992		1			1		1		1		4
1993		1			1				1		3
1994	1				3		1		2		7
1995	1				2		1				4
1996					1		1			1	3
1997	3						2				5
1998									4		4
1999					3		1		1		5
2000					3		1				4
2001					1	2			2		5
2002					2			1	1		4
2003					2	1					3
2004					3		1		1		5
2005	2			2	17		3		3	3	30
2006				1	13	2	1	3		3	23
2007				1	7	3	1		1		13
2008				5	4	1	5		8	1	24
2009	1			1				7	2		11
2010				1	9	1	1	2	1		15
2011	2				5			1	1	3	12
2012				5	3		1		1		10
2013					5		1	2	2	1	11
2014			2	2	6	1				1	12
2015	1		1	2	8	1	1		4		18
2016	1			2	7	1	1	1	2	2	17
2017				3	10			2			15
**Total**	**13**	**2**	**3**	**25**	**118**	**14**	**25**	**19**	**38**	**15**	**272**

**Table 3:** Summary of the numbers of EHV-1 isolates genotyped.

Disease Expression	Isolates	Horses	Outbreaks	Premises ^2^	Multi-Locus Typing	ORF68 Typing
Respiratory	3	3	3	3	3	3
Single abortion/ neonatal foal death	171	170 ^1^	170	163	171	164
Multiple abortion/ neonatal foal death	73	71	43	42	73	38
Single neurological disease	11	11 ^1^	11	11	11	7
Multiple neurological disease	14	14	11	11	14	10
Total	272	269	238	220 ^2^	272	222

^1^ One mare is categorised under single abortion and single neurological disease expression. Isolate IRL/766/2008 was collected when she exhibited neurological signs and isolate IRL/155/2008 was collected from her aborted foetus 11 weeks later. ^2^ Some premises experienced EHV-1 outbreaks in different years therefore the total number of outbreaks.

**Table 4:** Non-synonymous sites between EHV-1 Ab4 and V592 used for multi-locus analysis.

		AA Variation		
ORF	AA Position ^1^	Ab4	V592	Other ^2^
2	59	G	D	/^3^
5	114	G	V	/
8	114	D	N	/
11	189	Q	K	/
11	235	R	R	M
13	305	S	L	/
13	405	A	A	T
13	460	A	T	/
13	492	E	E	K
13	493	T	T	I
13	499	A	A	T
14	618–620	-^4^	PSR	/
14	628	R	R	K
14	692	S	S	N
15	166	D	N	/
22	430	S	P	/
29	12	T	K	/
30	752	D	N	/
30	990	E	K	/
31	90	N	S	/
32	42	S	L	/
33	15	N	H	/
33	976	N	D	/
34	66	D	G	/
36	47	S	R	/
37	265	A	V	/
39	440	S	L	/
40	196	R	H	/
42	1275	K	R	/
45	427	E	G	/
46	140	F	S	/
50	367	P	S	/
52	386	A	V	/
57	804	K	R	/
73	122	A	V	/
76	128	F	S	/

^1^ Amino acid positions are numbered according to V592 amino acid sequence. ^2^ Refers to additional amino acid coding changes observed in amino acid alignments of other EHV-1 strains. ^3^ Not applicable. ^4^ Gap in sequence.

**Table 5:** Primers used for ORF68 amplification and sequencing.

Primer	Description	Primer Sequence 5′ to 3′	Nucleotide Position on Strain Ab4
Forward	PCR	ATGGGTGTGGTCTTAATTAC	126275–126256
Reverse	PCR	GACACCGCCTGAAGTAGGAG	124963–124982
68R2	Sequencing	ACCGTTGAGCATAATCATCC [21]	125730–125710
68S1	Sequencing	GAAGATAGAATGGGTGTGAG [21]	125999–125979

## Supplementary Materials

The following are available online at http://www.mdpi.com/2076-0817/8/1/7/s1, Figure S1: Alignment of 322 EHV-1 artificial peptide sequences; Table S1: Summary of EHV-1 strains genotyped and listed in order of U_L_ clades; Table S2: Accession codes for EHV-1 sequences used in alignment; Table S3: Premises where more than one isolate was characterised by multi-locus sequence typing; Table S4: Details of EHV-1 ORF68 sequences used in network analysis; Table S5: Primer sequences used for multi-locus amplification.

## References

[1] Lunn D.P., Davis-Poynter N., Flaminio M.J., Horohov D.W., Osterrieder K., Pusterla N., Townsend H.G. (2009). Equine herpesvirus-1 consensus statement. J. Vet. Intern. Med..

[2] Davison A.J., Eberle R., Ehlers B., Hayward G.S., McGeoch D.J., Minson A.C., Pellett P.E., Roizman B., Studdert M.J., Thiry E. (2009). The order Herpesvirales. Arch. Virol..

[3] Crowhurst F.A., Dickinson G., Burrows R. (1981). An outbreak of paresis in mares and geldings associated with equid herpesvirus 1. Vet. Rec..

[4] McCartan C.G., Russell M.M., Wood J.L., Mumford J.A. (1995). Clinical, serological and virological characteristics of an outbreak of paresis and neonatal foal disease due to equine herpesvirus-1 on a stud farm. Vet. Rec..

[5] Mumford J.A., Edington N. (1980). EHV1 and equine paresis. Vet. Rec..

[6] Whitwell K.E., Blunden A.S. (1992). Pathological findings in horses dying during an outbreak of the paralytic form of Equid herpesvirus type 1 (EHV-1) infection. Equine Vet. J..

[7] Dayaram A., Franz M., Schattschneider A., Damiani A.M., Bischofberger S., Osterrieder N., Greenwood A.D. (2017). Long term stability and infectivity of herpesviruses in water. Sci. Rep..

[8] Patel J.R., Heldens J. (2005). Equine herpesviruses 1 (EHV-1) and 4 (EHV-4)—Epidemiology, disease and immunoprophylaxis: A brief review. Vet. J..

[9] Kydd J.H., Smith K.C., Hannant D., Livesay G.J., Mumford J.A. (1994). Distribution of equid herpesvirus-1 (EHV-1) in respiratory tract associated lymphoid tissue: Implications for cellular immunity. Equine Vet. J..

[10] Smith D.J., Hamblin A.S., Edington N. (2001). Infection of endothelial cells with equine herpesvirus-1 (EHV-1) occurs where there is activation of putative adhesion molecules: A mechanism for transfer of virus. Equine Vet. J..

[11] Edington N., Bridges C.G., Patel J.R. (1986). Endothelial cell infection and thrombosis in paralysis caused by equid herpesvirus-1: Equine stroke. Arch. Virol..

[12] USDA-APHIS 2007 Equine Herpesvirus Myeloencephalopathy: A Potentially Emerging Disease. http://www.aphis.usda.gov/vs/ceah/cei/taf/emergingdiseasenoticefiles/EHV-1final.pdf.

[13] OIE OIE-Listed Diseases, Infections and Infestations in Force in 2018. http://www.oie.int/animal-health-in-the-world/oie-listed-diseases-2018/.

[14] Telford E.A., Watson M.S., McBride K., Davison A.J. (1992). The DNA sequence of equine herpesvirus-1. Virology.

[15] Shakya A.K., O’Callaghan D.J., Kim S.K. (2017). Comparative Genomic Sequencing and Pathogenic Properties of Equine Herpesvirus 1 KyA and RacL11. Front. Vet. Sci..

[16] Mumford J.A., Hannant D., Jessett D.M., O’Neill T., Smith K.C., Ostlund E.N. Abortigenic and neurological disease caused by experimental infection with equid herpesvirus-1. Proceedings of the Seventh International Conference on Equine Infectious Diseases.

[17] Gardiner D.W., Lunn D.P., Goehring L.S., Chiang Y.W., Cook C., Osterrieder N., McCue P., Del Piero F., Hussey S.B., Hussey G.S. (2012). Strain impact on equine herpesvirus type 1 (EHV-1) abortion models: Viral loads in fetal and placental tissues and foals. Vaccine.

[18] Smith K.C., Whitwell K.E., Binns M.M., Dolby C.A., Hannant D., Mumford J.A. (1992). Abortion of virologically negative foetuses following experimental challenge of pregnant pony mares with equid herpesvirus 1. Equine Vet. J..

[19] Smith K.C., Whitwell K.E., Mumford J.A., Hannant D., Blunden A.S., Tearle J.P. (2000). Virulence of the V592 isolate of equid herpesvirus-1 in ponies. J. Comp. Pathol..

[20] Mumford J.A., Rossdale P.D., Jessett D.M., Gann S.J., Ousey J., Cook R.F. (1987). Serological and virological investigations of an equid herpesvirus 1 (EHV-1) abortion storm on a stud farm in 1985. J. Reprod. Fertil. Suppl..

[21] Nugent J., Birch-Machin I., Smith K.C., Mumford J.A., Swann Z., Newton J.R., Bowden R.J., Allen G.P., Davis-Poynter N. (2006). Analysis of equid herpesvirus 1 strain variation reveals a point mutation of the DNA polymerase strongly associated with neuropathogenic versus nonneuropathogenic disease outbreaks. J. Virol..

[22] Goodman L.B., Loregian A., Perkins G.A., Nugent J., Buckles E.L., Mercorelli B., Kydd J.H., Palu G., Smith K.C., Osterrieder N. (2007). A point mutation in a herpesvirus polymerase determines neuropathogenicity. PLoS Pathog..

[23] Van de Walle G.R., Goupil R., Wishon C., Damiani A., Perkins G.A., Osterrieder N. (2009). A single-nucleotide polymorphism in a herpesvirus DNA polymerase is sufficient to cause lethal neurological disease. J. Infect. Dis..

[24] Cuxson J.L., Hartley C.A., Ficorilli N.P., Symes S.J., Devlin J.M., Gilkerson J.R. (2014). Comparing the genetic diversity of ORF30 of Australian isolates of 3 equid alphaherpesviruses. Vet. Microbiol..

[25] Fritsche A.K., Borchers K. (2011). Detection of neuropathogenic strains of Equid Herpesvirus 1 (EHV-1) associated with abortions in Germany. Vet. Microbiol..

[26] Perkins G.A., Goodman L.B., Tsujimura K., Van de Walle G.R., Kim S.G., Dubovi E.J., Osterrieder N. (2009). Investigation of the prevalence of neurologic equine herpes virus type 1 (EHV-1) in a 23-year retrospective analysis (1984–2007). Vet. Microbiol..

[27] Pronost S., Cook R.F., Fortier G., Timoney P.J., Balasuriya U.B.R. (2010). Relationship between equine herpesvirus-1 myeloencephalopathy and viral genotype. Equine Vet. J..

[28] Meindl A., Osterrieder N. (1999). The equine herpesvirus 1 Us2 homolog encodes a nonessential membrane-associated virion component. J. Virol..

[29] Anagha G., Gulati B.R., Riyesh T., Virmani N. (2017). Genetic characterization of equine herpesvirus 1 isolates from abortion outbreaks in India. Arch. Virol..

[30] Malik P., Balint A., Dan A., Palfi V. (2012). Molecular characterisation of the ORF68 region of equine herpesvirus-1 strains isolated from aborted fetuses in Hungary between 1977 and 2008. Acta Vet. Hung..

[31] Negussie H., Gizaw D., Tessema T.S., Nauwynck H.J. (2017). Equine Herpesvirus-1 Myeloencephalopathy, an Emerging Threat of Working Equids in Ethiopia. Transbound. Emerg. Dis..

[32] Stasiak K., Dunowska M., Hills S.F., Rola J. (2017). Genetic characterization of equid herpesvirus type 1 from cases of abortion in Poland. Arch. Virol..

[33] Tsujimura K., Oyama T., Katayama Y., Muranaka M., Bannai H., Nemoto M., Yamanaka T., Kondo T., Kato M., Matsumura T. (2011). Prevalence of Equine Herpesvirus Type 1 Strains of Neuropathogenic Genotype in a Major Breeding Area of Japan. J. Vet. Med. Sci..

[34] Vaz P.K., Horsington J., Hartley C.A., Browning G.F., Ficorilli N.P., Studdert M.J., Gilkerson J.R., Devlin J.M. (2016). Evidence of widespread natural recombination among field isolates of equine herpesvirus 4 but not among field isolates of equine herpesvirus 1. J. Gen. Virol..

[35] Bryant N.A., Wilkie G.S., Russell C.A., Compston L., Grafham D., Clissold L., McLay K., Medcalf L., Newton R., Davison A.J. (2018). Genetic diversity of equine herpesvirus 1 isolated from neurological, abortigenic and respiratory disease outbreaks. Transbound. Emerg. Dis..

[36] Loncoman C.A., Vaz P.K., Coppo M.J.C., Hartley C.A., Morera F.J., Browning G.F., Devlin J.M. (2017). Natural recombination in alphaherpesviruses: Insights into viral evolution through full genome sequencing and sequence analysis. Infect. Genet. Evol..

[37] Pagamjav O., Sakata T., Matsumura T., Yamaguchi T., Fukushi H. (2005). Natural recombinant between equine herpesviruses 1 and 4 in the ICP4 gene. Microbiol. Immunol..

[38] Greenwood A.D., Tsangaras K., Ho S.Y., Szentiks C.A., Nikolin V.M., Ma G., Damiani A., East M.L., Lawrenz A., Hofer H. (2012). A potentially fatal mix of herpes in zoos. Curr. Biol. CB.

[39] Jensen N.J., Rivailler P., Tseng H.F., Quinlivan M.L., Radford K., Folster J., Harpaz R., LaRussa P., Jacobsen S., Scott Schmid D. (2017). Revisiting the genotyping scheme for varicella-zoster viruses based on whole-genome comparisons. J. Gen. Virol..

[40] Norberg P. (2010). Divergence and genotyping of human alpha-herpesviruses: An overview. Infect. Genet. Evol. J. Mol. Epidemiol. Evol. Genet. Infect. Dis..

[41] Houldcroft C.J., Beale M.A., Breuer J. (2017). Clinical and biological insights from viral genome sequencing. Nat. Rev. Microbiol..

[42] Allen G.P. (2007). Development of a real-time polymerase chain reaction assay for rapid diagnosis of neuropathogenic strains of equine herpesvirus-1. J. Vet. Diagn. Investig..

[43] Smith K.L., Li Y., Breheny P., Cook R.F., Henney P.J., Sells S., Pronost S., Lu Z., Crossley B.M., Timoney P.J. (2012). New Real-Time PCR Assay Using Allelic Discrimination for Detection and Differentiation of Equine Herpesvirus-1 Strains with A(2254) and G(2254) Polymorphisms. J. Clin. Microbiol..

[44] Burgess B.A., Tokateloff N., Manning S., Lohmann K., Lunn D.P., Hussey S.B., Morley P.S. (2012). Nasal shedding of equine herpesvirus-1 from horses in an outbreak of equine herpes myeloencephalopathy in Western Canada. J. Vet. Intern. Med..

[45] Franz M., Goodman L.B., Van de Walle G.R., Osterrieder N., Greenwood A.D. (2017). A Point Mutation in a Herpesvirus Co-Determines Neuropathogenicity and Viral Shedding. Viruses.

[46] Holz C.L., Nelli R.K., Wilson M.E., Zarski L.M., Azab W., Baumgardner R., Osterrieder N., Pease A., Zhang L., Hession S. (2017). Viral genes and cellular markers associated with neurological complications during herpesvirus infections. J. Gen. Virol..

[47] Allen G.P. (2008). Risk factors for development of neurologic disease after experimental exposure to equine herpesvirus-1 in horses. Am. J. Vet. Res..

[48] Smith K.L., Allen G.P., Branscum A.J., Frank Cook R., Vickers M.L., Timoney P.J., Balasuriya U.B. (2010). The increased prevalence of neuropathogenic strains of EHV-1 in equine abortions. Vet. Microbiol..

[49] Pronost S., Legrand L., Pitel P.H., Wegge B., Lissens J., Freymuth F., Richard E., Fortier G. (2012). Outbreak of equine herpesvirus myeloencephalopathy in France: A clinical and molecular investigation. Transbound. Emerg. Dis..

[50] Kydd J.H., Slater J., Osterrieder N., Lunn D.P., Antczak D.F., Azab W., Balasuriya U.B., Barnett C., Brosnahan M., Cook C. (2012). Third International Havemeyer Workshop on Equine Herpesvirus type 1. Equine Vet. J..

[51] Stasiak K., Rola J., Ploszay G., Socha W., Zmudzinski J.F. (2015). Detection of the neuropathogenic variant of equine herpesvirus 1 associated with abortions in mares in Poland. BMC Vet. Res..

[52] Vissani M.A., Becerra M.L., Olguin Perglione C., Tordoya M.S., Mino S., Barrandeguy M. (2009). Neuropathogenic and non-neuropathogenic genotypes of Equid Herpesvirus type 1 in Argentina. Vet. Microbiol..

[53] Edington N., Smyth B., Griffiths L. (1991). The role of endothelial cell infection in the endometrium, placenta and foetus of equid herpesvirus 1 (EHV-1) abortions. J. Comp. Pathol..

[54] Allen G.P., Breathnach C.C. (2006). Quantification by real-time PCR of the magnitude and duration of leucocyte-associated viraemia in horses infected with neuropathogenic vs. non-neuropathogenic strains of EHV-1. Equine Vet. J..

[55] Vandekerckhove A.P., Glorieux S., Gryspeerdt A.C., Steukers L., Duchateau L., Osterrieder N., Van de Walle G.R., Nauwynck H.J. (2010). Replication kinetics of neurovirulent versus non-neurovirulent equine herpesvirus type 1 strains in equine nasal mucosal explants. J. Gen. Virol..

[56] Barbic L., Lojkic I., Stevanovic V., Bedekovic T., Staresina V., Lemo N., Lojkic M., Madic J. (2012). Two outbreaks of neuropathogenic equine herpesvirus type 1 with breed-dependent clinical signs. Vet. Rec..

[57] Damiani A.M., de Vries M., Reimers G., Winkler S., Osterrieder N. (2014). A severe equine herpesvirus type 1 (EHV-1) abortion outbreak caused by a neuropathogenic strain at a breeding farm in northern Germany. Vet. Microbiol..

[58] Walter J., Seeh C., Fey K., Bleul U., Osterrieder N. (2013). Clinical observations and management of a severe equine herpesvirus type 1 outbreak with abortion and encephalomyelitis. Acta Vet. Scand..

[59] McFadden A.M., Hanlon D., McKenzie R.K., Gibson I., Bueno I.M., Pulford D.J., Orr D., Dunowska M., Stanislawek W.L., Spence R.P. (2016). The first reported outbreak of equine herpesvirus myeloencephalopathy in New Zealand. N. Z. Vet. J..

[60] Pronost S., Léon A., Legrand L., Fortier C., Miszczak F., Freymuth F., Fortier G. (2010). Neuropathogenic and non-neuropathogenic variants of equine herpesvirus 1 in France. Vet. Microbiol..

[61] Goehring L.S., van Winden S.C., van Maanen C., Sloet van Oldruitenborgh-Oosterbaan M.M. (2006). Equine herpesvirus type 1-associated myeloencephalopathy in The Netherlands: A four-year retrospective study (1999–2003). J. Vet. Intern. Med..

[62] Brosnahan M.M., Al Abri M.A., Brooks S.A., Antczak D.F., Osterrieder N. (2018). Genome-wide association study of equine herpesvirus type 1-induced myeloencephalopathy identifies a significant single nucleotide polymorphism in a platelet-related gene. Vet. J..

[63] Gryspeerdt A., Vanderkerckhove A., Van Doorsselaere J., Van de Walle G., Nauwynck H. (2011). Description of an unusually large outbreak of nervous system disorders caused by equine herpesvirus 1 (EHV1) in 2009 in Belgium. Vlaams Diergeneeskundig Tijdschrif.

[64] Irish Thoroughbred Marketing. http://www.itm.ie.

[65] Allen G.P., Kydd J.H., Slater J.D., Smith K.C. (2004). Equid Herpesvirus 1 and Equid Herpesvirus 4 Infections. Infectious Diseaases of Livestock.

[66] Larkin M.A., Blackshields G., Brown N.P., Chenna R., McGettigan P.A., McWilliam H., Valentin F., Wallace I.M., Wilm A., Lopez R. (2007). Clustal W and Clustal X version 2.0. Bioinformatics.

[67] Hall T.A. (1999). BioEdit: A user-friendly biological sequence alignment editor and analysis program for Windows 95/98/NT. Nucleic Acids Symp. Ser..

[68] Benson D.A., Cavanaugh M., Clark K., Karsch-Mizrachi I., Lipman D.J., Ostell J., Sayers E.W. (2013). GenBank. Nucleic Acids Res..

[69] Edgar R.C. (2004). MUSCLE: Multiple sequence alignment with high accuracy and high throughput. Nucleic Acids Res..

[70] Kumar S., Stecher G., Tamura K. (2016). MEGA7: Molecular Evolutionary Genetics Analysis Version 7.0 for Bigger Datasets. Mol. Biol. Evol..

[71] Jones D.T., Taylor W.R., Thornton J.M. (1992). The rapid generation of mutation data matrices from protein sequences. Comput. Appl. Biosci. CABIOS.

[72] Rozen S., Skaletsky H. (2000). Primer3 on the WWW for general users and for biologist programmers. Methods Mol. Biol..

[73] Rice P., Longden I., Bleasby A. (2000). EMBOSS: The European Molecular Biology Open Software Suite. Trends Genet. TIG.

[74] Leigh J., Bryant D. (2015). PopART: Full-Feature Software for Haplotype Network Construction. Methods Ecol Evol..

