# Supplementary material for: Molecular Characterisation of Equine Herpesvirus 1 Isolates from Cases of Abortion, Respiratory and Neurological Disease in Ireland between 1990 and 2017
Source: Pathogens. 2019 Jan 15;8(1):7. doi: 10.3390/pathogens8010007 (PMC6471309; doi:10.3390/pathogens8010007)
Supplement: Supplementary file 1 [file pathogens-08-00007-s001.zip › SupplementaryData/Supplementary Figure S1.docx]

Supplementary Figure S1. Alignment of 322 EHV-1 artificial peptide sequences

10 20 30

....|....|....|....|....|....|....|...

**EHV-1.Ab4**  **GGDQRSAAETA---RSDSTDENSNNDSASRKEFPAKAF** 35

**LEICES/22/1996**  **...........---........................** 35

**UK/32/1982**  **...........---........................** 35

**HERTS/188/2010**  **...........---........................** 35

**EHV-1.HH1**  **D..........---.....N..................** 35

**HONG KONG/57/1984** **D..........---.....N..................** 35

**EHV-1.1074-94**  **D..........PSR.....N..................** 38

**BERKS/7/1996**  **D..........PSR.....N..................** 38

**HAMP/1/2008**  **D..........PSR.....N..................** 38

**EHV-1.NY03**  **D..........PSR.....N..................** 38

**IRL/164/1994**  **D..........---........................** 35

**IRL/443/2005**  **D..........---........................** 35

**IRL/710/1990**  **D..........---........................** 35

**IRL/325/1995**  **D..........---........................** 35

**IRL/440/2005**  **D..........---........................** 35

**IRL/497/1997**  **...........---........................** 35

**IRL/584/1997**  **...........---........................** 35

**IRL/559/2009**  **D..........---.....N..................** 35

**ITA/055/2011**  **D..........---.....N..................** 35

**ITA/056/2011**  **D..........---.....N..................** 35

**IRL/600/1997**  **D..........---.....N..................** 35

**IRL/366/2016**  **D..........PSR.....N..................** 38

**IRL/282/2015**  **D..........PSR........................** 38

**EHV-1.00C19**  **D..........PSR.....N.................S** 38

**EHV-1.3045-07**  **D..........PSR.....N.................S** 38

**EHV-1.717A-82**  **D..........PSR.....N.................S** 38

**EHV-1.89C105**  **D..........PSR.....N.................S** 38

**EHV-1.89C25**  **D..........PSR.....N.................S** 38

**EHV-1.01C1**  **D..........PSR.........D.............S** 38

**EHV-1.90C16**  **D..........PSR.....N...D.............S** 38

**IRL/154/1993**  **D......T...PSR..........D..........R.S** 38

**IRL/962/1992**  **D..........PSR.....N.................S** 38

**SUFF/123/2005**  **D..........PSR.....N....D..V...GS....S** 38

**IRL/127/2005**  **D..........PSR.....N....D..V...GS....S** 38

**IRL/894/2005**  **D..........PSR.....N....D..V...GS....S** 38

**IRL/383/2005**  **D..........PSR.....N....D..V...GS....S** 38

**IRL/847/2006**  **D..........PSR.....N....D..V...GS....S** 38

**IRL/079/2006**  **D..........PSR.....N....D..V...GS....S** 38

**IRL/411/2006**  **D..........PSR.....N....D..V...GS....S** 38

**IRL/003/2008**  **D..........PSR.....N....D..V...GS....S** 38

**IRL/741/1996**  **D..........PSR.....N....D..V...GS....S** 38

**IRL/099/2011**  **D..........PSR.....N....D..V...GS....S** 38

**IRL/276/2013**  **D..........PSR.....N....D..V...GS....S** 38

**IRL/912/2014**  **D..........PSR.....N....D..V...GS....S** 38

**IRL/791/2016**  **D..........PSR.....N....D..V...GS....S** 38

**IRL/526/2016**  **D..........PSR.....N....D..V...GS....S** 38

**IRL/331/2011**  **D..........PSR..........D..V...GS....S** 38

**IRL/350/2011**  **D..........PSR..........D..V...GS....S** 38

**OXFORD/206/2013**  **D..........PSR..N..N...HD..V...GS.V..S** 38

**EHV-1.OH03**  **D..........PSR..N......HD..V...GS.V..S** 38

**EHV-1.T953**  **D..........PSR..N......HD..V...GS.V..S** 38

**IRL/916/2014**  **D..........PSR..N..N...HD..V...GS.V..S** 38

**Supplementary Figure S1 (Continued)**

**IRL/546/2014**  **D..........PSR..N..N...HD..V...GS.V..S** 38

**IRL/801/2015**  **D..........PSR..N..N...HD..V...GS.V..S** 38

**EHV-1.2222-03**  **D.....T....PSR..N..N...HD..V...GS.V..S** 38

**EHV-1.NZA-77**  **D.....T....PSR..N..N...HD..V...GS.V..S** 38

**EHV-1.VA02**  **D.....T....PSR..N..N...HD..V...GS.V..S** 38

**EHV-1.NY05**  **D..........PSR..N......HD..V...GS.V..S** 38

**EHV-1.1029-93**  **D..........PSR..N..N...HD..V...GS.V..S** 38

**SUFF/10/2012**  **D.....T.K..PSR..N..N...HD..V...GS.V..S** 38

**IRL/919/2005**  **D.....T.K..PSR..N..N...HD..V...GS.V..S** 38

**IRL/025/2005**  **D.....T.K..PSR..N..N...HD..V...GS.V..S** 38

**IRL/130/2006**  **D.....T.K..PSR..N..N...HD..V...GS.V..S** 38

**IRL/837/2007**  **D.....T.K..PSR..N..N...HD..V...GS.V..S** 38

**IRL/177/2008**  **D.....T.K..PSR..N..N...HD..V...GS.V..S** 38

**IRL/307/2008**  **D.....T.K..PSR..N..N...HD..V...GS.V..S** 38

**IRL/238/2008**  **D.....T.K..PSR..N..N...HD..V...GS.V..S** 38

**IRL/766/2008**  **D.....T.K..PSR..N..N...HD..V...GS.V..S** 38

**IRL/155/2008**  **D.....T.K..PSR..N..N...HD..V...GS.V..S** 38

**IRL/671/2009**  **D.....T.K..PSR..N..N...HD..V...GS.V..S** 38

**IRL/721/2010**  **D.....T.K..PSR..N..N...HD..V...GS.V..S** 38

**IRL/169/2012**  **D.....T.K..PSR..N..N...HD..V...GS.V..S** 38

**IRL/570/2012**  **D.....T.K..PSR..N..N...HD..V...GS.V..S** 38

**IRL/572/2012**  **D.....T.K..PSR..N..N...HD..V...GS.V..S** 38

**IRL/863/2012**  **D.....T.K..PSR..N..N...HD..V...GS.V..S** 38

**IRL/948/2012**  **D.....T.K..PSR..N..N...HD..V...GS.V..S** 38

**IRL/159/2014**  **D.....T.K..PSR..N..N...HD..V...GS.V..S** 38

**IRL/031/2014**  **D.....T.K..PSR..N..N...HD..V...GS.V..S** 38

**IRL/983/2015**  **D.....T.K..PSR..N..N...HD..V...GS.V..S** 38

**IRL/050/2015**  **D.....T.K..PSR..N..N...HD..V...GS.V..S** 38

**IRL/784/2016**  **D.....T.K..PSR..N..N...HD..V...GS.V..S** 38

**IRL/704/2016**  **D.....T.K..PSR..N..N...HD..V...GS.V..S** 38

**IRL/425/2017**  **D.....T.K..PSR..N..N...HD..V...GS.V..S** 38

**IRL/426/2017**  **D.....T.K..PSR..N..N...HD..V...GS.V..S** 38

**IRL/001/2017**  **D.....T.K..PSR..N..N...HD..V...GS.V..S** 38

**LINCS/2/2012**  **D.....T.K..PSR..N..N...HD..V...GS.V..S** 38

**OXFORD/27/2011**  **D......T..TPSRKNN......HD..V...GS.V..S** 38

**EHV-1.NMKT04**  **D......T..TPSRKNN.....LHD..V...GS.V..S** 38

**IRL/268/2001**  **D......T..TPSRKNN......HD..V...GS.V..S** 38

**IRL/634/2001**  **D......T..TPSRKNN......HD..V...GS.V..S** 38

**IRL/184/2006**  **D......T..TPSRKNN......HD..V...GS.V..S** 38

**IRL/306/2006**  **D......T..TPSRKNN......HD..V...GS.V..S** 38

**IRL/642/2007**  **D......T..TPSRKNN......HD..V...GS.V..S** 38

**IRL/961/2007**  **D......T..TPSRKNN......HD..V...GS.V..S** 38

**IRL/568/2007**  **D......T..TPSRKNN......HD..V...GS.V..S** 38

**IRL/995/2008**  **D......T..TPSRKNN......HD..V...GS.V..S** 38

**IRL/612/2010**  **D......T..TPSRKNN......HD..V...GS.V..S** 38

**IRL/218/2014**  **D......T..TPSRKNN......HD..V...GS.V..S** 38

**IRL/439/2015**  **D......T..TPSRKNN......HD..V...GS.V..S** 38

**IRL/917/2016**  **D......T..TPSRKNN......HD..V...GS.V..S** 38

**ITA/944/2003**  **D......T..TPSRKNN......HD..V...GS.V..S** 38

**IRL/542/1990**  **D......T..TPSRKNN....S.HD..V......V..S** 38

**SUFF/125/2013**  **D...M..T...PSRKNN..N.S.HD..V...GS.VR.S** 38

**SUFF/48/2013**  **D...M..T...PSRKNN..N.S.HD..V...GS.VR.S** 38

**EHV-1.196-02**  **D...M..T...PSRKNN..N.S.HD..V...GS.VR.S** 38

**Supplementary Figure S1 (Continued)**

**EHV-1.2019-02**  **D...M..T...PSRKNN..N.S.HD..V...GS.VR.S** 38

**EHV-1.3038-07**  **D...M..T...PSRKNN..N.S.HD..V...GS.VR.S** 38

**EHV-1.438-77**  **D...M..T...PSRKNN..N.S.HD..V...GS.VR.S** 38

**EHV-1.970-90**  **D...M..T...PSRKNN..N.S.HD..V...GS.VR.S** 38

**EHV-1.FL06**  **D...M..T...PSRKNN....S.HD..V...GS.VR.S** 38

**IRL/461/2002**  **D...M..T...PSRKNN....S.HD..V...GS.VR.S** 38

**IRL/048/1994**  **D...M..T...---KNN....S.HD..V...GS.VR.S** 35

**IRL/991/2016**  **D...M..T...PSRKNN....S.HD..V...GS.VR.S** 38

**IRL/441/2000**  **D...M..T...PSRKNN....S.HD..V...GS.VR.S** 38

**IRL/573/2017**  **D...M..T...PSRKNN....S.HD..V...GS.VR.S** 38

**IRL/998/2005**  **D...M..T...PSRKNN..N.S.HD..V...GS.VR.S** 38

**IRL/739/2015**  **D...M..T...PSRKNN..N.S.HD..V...GS.VR.S** 38

**IRL/694/2014**  **D...M..T...PSRKNN..N.S.HD..V...GS.VR.S** 38

**IRL/212/1999**  **D...M..T...PSRKNN..N.S.HD..V...GS.VR.S** 38

**IRL/410/2008**  **D...M..T...PSRKNN..N.S.HD..V...GS.VR.S** 38

**IRL/617/2016**  **D...M..T...PSRKNN..N.S.HD..V...GS.VR.S** 38

**IRL/948/2017**  **D...M..T...PSRKNN..N.S.HD..V...GS.VR.S** 38

**IRL/212/1993**  **D...M..T...PSRKNN..N.S.HD..V...GS.VR.S** 38

**IRL/279/2005**  **D...M..T...PSRKNN..N.S.HD..V...GS.VR.S** 38

**IRL/987/2015**  **D...M..T...PSRKNN..N.S.HD..V...GS.VR.S** 38

**IRL/332/2017**  **D...M..T...PSRKNN..N.S.HD..V...GS.VR.S** 38

**IRL/952/2005**  **D...M..T...PSRKNN..N.S.HD..V...GS.VR.S** 38

**IRL/943/2006**  **D...M..T...PSRKNN..N.S.HD..V...GS.VR.S** 38

**IRL/368/2008**  **D...M..T...PSRKNN..N.S.HD..V...GS.VR.S** 38

**IRL/967/2014**  **D...M..T...PSRKNN..N.S.HD..V...GS.VR.S** 38

**IRL/820/2006**  **D...M..T...PSRKNN..N.S.HD..V...GS.VR.S** 38

**IRL/315/2006**  **D...M..T...PSRKNN..N.S.HD..V...GS.VR.S** 38

**IRL/374/2006**  **D...M..T...PSRKNN..N.S.HD..V...GS.VR.S** 38

**IRL/935/2006**  **D...M..T...PSRKNN..N.S.HD..V...GS.VR.S** 38

**IRL/463/1999**  **D...M..T...PSRKNN..N.S.HD..V...GS.VR.S** 38

**IRL/923/2011**  **D...M..T...PSRKNN..N.S.HD..V...GS.VR.S** 38

**IRL/905/1991**  **D...M..T...PSRKNN..N.S.HD..V...GS.VR.S** 38

**IRL/064/1994**  **D...M..T...PSRKNN..N.S.HD..V...GS.VR.S** 38

**IRL/609/1995**  **D...M..T...PSRKNN..N.S.HD..V...GS.VR.S** 38

**IRL/096/1996**  **D...M..T...PSRKNN..N.S.HD..V...GS.VR.S** 38

**IRL/813/2003**  **D...M..T...PSRKNN..N.S.HD..V...GS.VR.S** 38

**IRL/942/2004**  **D...M..T...PSRKNN..N.S.HD..V...GS.VR.S** 38

**IRL/094/2005**  **D...M..T...PSRKNN..N.S.HD..V...GS.VR.S** 38

**IRL/559/2006**  **D...M..T...PSRKNN..N.S.HD..V...GS.VR.S** 38

**IRL/060/2008**  **D...M..T...PSRKNN..N.S.HD..V...GS.VR.S** 38

**IRL/241/2012**  **D...M..T...PSRKNN..N.S.HD..V...GS.VR.S** 38

**IRL/055/2005**  **D...M..T...PSRKNN..N.S.HD..V...GS.VR.S** 38

**IRL/108/2005**  **D...M..T...PSRKNN..N.S.HD..V...GS.VR.S** 38

**IRL/776/1995**  **D...M..T...PSRKNN..N.S.HD..V...GS.VR.S** 38

**IRL/541/2005**  **D...M..T...PSRKNN..N.S.HD..V...GS.VR.S** 38

**IRL/123/2006**  **D...M..T...PSRKNN..N.S.HD..V...GS.VR.S** 38

**IRL/114/2010**  **D...M..T...PSRKNN..N.S.HD..V...GS.VR.S** 38

**IRL/332/2013**  **D...M..T...PSRKNN..N.S.HD..V...GS.VR.S** 38

**IRL/061/2016**  **D...M..T...PSRKNN..N.S.HD..V...GS.VR.S** 38

**IRL/596/2017**  **D...M..T...PSRKNN..N.S.HD..V...GS.VR.S** 38

**IRL/396/2006**  **D...M..T...PSRKNN..N.S.HD..V...GS.VR.S** 38

**IRL/745/2011**  **D...M..T...PSRKNN..N.S.HD..V...GS.VR.S** 38

**IRL/433/2014**  **D...M..T...PSRKNN..N.S.HD..V...GS.VR.S** 38

**Supplementary Figure S1 (Continued)**

**IRL/197/2017**  **D...M..T...PSRKNN..N.S.HD..V...GS.VR.S** 38

**IRL/694/2005**  **D...M..T...PSRKNN..N.S.HD..V...GS.VR.S** 38

**IRL/979/2007**  **D...M..T...PSRKNN..N.S.HD..V...GS.VR.S** 38

**IRL/902/2011**  **D...M..T...PSRKNN..N.S.HD..V...GS.VR.S** 38

**IRL/280/2016**  **D...M..T...PSRKNN..N.S.HD..V...GS.VR.S** 38

**IRL/206/1994**  **D...M..T...PSRKNN..N.S.HD..V...GS.VR.S** 38

**IRL/898/2007**  **D...M..T...PSRKNN..N.S.HD..V...GS.VR.S** 38

**IRL/569/2008**  **D...M..T...PSRKNN..N.S.HD..V...GS.VR.S** 38

**IRL/749/2014**  **D...M..T...PSRKNN..N.S.HD..V...GS.VR.S** 38

**IRL/564/2015**  **D...M..T...PSRKNN..N.S.HD..V...GS.VR.S** 38

**IRL/024/2017**  **D...M..T...PSRKNN..N.S.HD..V...GS.VR.S** 38

**IRL/065/2005**  **D...M..T...PSRKNN..N.S.HD..V...GS.VR.S** 38

**IRL/670/2007**  **D...M..T...PSRKNN..N.S.HD..V...GS.VR.S** 38

**IRL/040/2016**  **D...M..T...PSRKNN..N.S.HD..V...GS.VR.S** 38

**IRL/071/2005**  **D...M..T...PSRKNN..N.S.HD..V...GS.VR.S** 38

**IRL/671/2007**  **D...M..T...PSRKNN..N.S.HD..V...GS.VR.S** 38

**IRL/052/2014**  **D...M..T...PSRKNN..N.S.HD..V...GS.VR.S** 38

**IRL/073/2005**  **D...M..T...PSRKNN..N.S.HD..V...GS.VR.S** 38

**IRL/895/2007**  **D...M..T...PSRKNN..N.S.HD..V...GS.VR.S** 38

**IRL/333/2014**  **D...M..T...PSRKNN..N.S.HD..V...GS.VR.S** 38

**IRL/125/2005**  **D...M..T...PSRKNN..N.S.HD..V...GS.VR.S** 38

**IRL/533/2006**  **D...M..T...PSRKNN..N.S.HD..V...GS.VR.S** 38

**IRL/926/2005**  **D...M..T...PSRKNN..N.S.HD..V...GS.VR.S** 38

**IRL/550/2015**  **D...M..T...PSRKNN..N.S.HD..V...GS.VR.S** 38

**IRL/904/2015**  **D...M..T...PSRKNN..N.S.HD..V...GS.VR.S** 38

**IRL/054/2005**  **D...M..T...PSRKNN..N.S.HD..V...GS.VR.S** 38

**IRL/017/1992**  **D...M..T...PSRKNN..N.S.HD..V...GS.VR.S** 38

**IRL/159/2000**  **D...M..T...PSRKNN..N.S.HD..V...GS.VR.S** 38

**IRL/311/2003**  **D...M..T...PSRKNN..N.S.HD..V...GS.VR.S** 38

**IRL/650/2004**  **D...M..T...PSRKNN..N.S.HD..V...GS.VR.S** 38

**IRL/775/2000**  **D...M..T...PSRKNN..N.S.HD..V...GS.VR.S** 38

**IRL/631/2010**  **D...M..T...PSRKNN..N.S.HD..V...GS.VR.S** 38

**IRL/409/2011**  **D...M..T...PSRKNN..N.S.HD..V...GS.VR.S** 38

**IRL/878/2015**  **D...M..T...PSRKNN..N.S.HD..V...GS.VR.S** 38

**IRL/356/2017**  **D...M..T...PSRKNN..N.S.HD..V...GS.VR.S** 38

**IRL/045/1999**  **D...M..T...PSRKNN..N.S.HD..V...GS.VR.S** 38

**IRL/467/2010**  **D...M..T...PSRKNN..N.S.HD..V...GS.VR.S** 38

**IRL/145/2017**  **D...M..T...PSRKNN..N.S.HD..V...GS.VR.S** 38

**IRL/481/2010**  **D...M..T...PSRKNN..N.S.HD..V...GS.VR.S** 38

**IRL/675/2016**  **D...M..T...PSRKNN..N.S.HD..V...GS.VR.S** 38

**IRL/067/2016**  **D...M..T...PSRKNN..N.S.HD..V...GS.VR.S** 38

**IRL/009/2002**  **D...M..T...PSRKNN..N.S.HD..V...GS.VR.S** 38

**IRL/882/2004**  **D...M..T...PSRKNN..N.S.HD..V...GS.VR.S** 38

**IRL/992/2005**  **D...M..T...PSRKNN..N.S.HD..V...GS.VR.S** 38

**IRL/539/2006**  **D...M..T...PSRKNN..N.S.HD..V...GS.VR.S** 38

**IRL/313/2010**  **D...M..T...PSRKNN..N.S.HD..V...GS.VR.S** 38

**IRL/013/2012**  **D...M..T...PSRKNN..N.S.HD..V...GS.VR.S** 38

**IRL/467/2017**  **D...M..T...PSRKNN..N.S.HD..V...GS.VR.S** 38

**IRL/074/2001**  **D...M..T...PSRKNN..N.S.HD..V...GS.VR.S** 38

**IRL/851/2010**  **D...M..T...PSRKNN..N.S.HD..V...GS.VR.S** 38

**IRL/222/2013**  **D...M..T...PSRKNN..N.S.HD..V...GS.VR.S** 38

**IRL/596/2015**  **D...M..T...PSRKNN..N.S.HD..V...GS.VR.S** 38

**IRL/726/2017**  **D...M..T...PSRKNN..N.S.HD..V...GS.VR.S** 38

**Supplementary Figure S1 (Continued)**

**IRL/130/2010**  **D...M..T...PSRKNN..N.S.HD..V...GS.VR.S** 38

**IRL/031/2012**  **D...M..T...PSRKNN..N.S.HD..V...GS.VR.S** 38

**IRL/223/2013**  **D...M..T...PSRKNN..N.S.HD..V...GS.VR.S** 38

**IRL/331/2010**  **D...M..T...PSRKNN..N.S.HD..V...GS.VR.S** 38

**IRL/534/2010**  **D...M..T...PSRKNN..N.S.HD..V...GS.VR.S** 38

**IRL/695/2006**  **D...M..T...PSRKNN..N.S.HD..V...GS.VR.S** 38

**IRL/795/2011**  **D...M..T...PSRKNN..N.S.HD..V...GS.VR.S** 38

**IRL/894/2013**  **D...M..T...PSRKNN..N.S.HD..V...GS.VR.S** 38

**IRL/558/2006**  **D...M..T...PSRKNN..N.S.HD..V...GS.VR.S** 38

**IRL/249/2013**  **D...M..T...PSRKNN..N.S.HD..V...GS.VR.S** 38

**IRL/001/1991**  **D...M..T...PSRKNN..N.S.HD..V...GS.VR.S** 38

**IRL/024/2005**  **D...M..T...PSRKNN..N.S.HD..V...GS.VR.S** 38

**IRL/824/2007**  **D...M..T...PSRKNN..N.S.HD..V...GS.VR.S** 38

**IRL/124/2005**  **D...M..T...PSRKNN..N.S.HD..V...GS.VR.S** 38

**IRL/754/2006**  **D...M..T...PSRKNN..N.S.HD..V...GS.VR.S** 38

**IRL/825/2007**  **D...M..T...PSRKNN..N.S.HD..V...GS.VR.S** 38

**IRL/001/2015**  **D...M..T...PSRKNN..N.S.HD..V...GS.VR.S** 38

**DEVON/28/2003**  **DV...L.T...PSR..N....S.HD..V...GS.VR.S** 38

**GLOUCS/127/1998**  **DV...L.T...PSR..N....S.HD..V...GS.VR.S** 38

**GLOUCS/54/2013**  **DV...L.T...PSR..N....S.HD..V...GS.VR.S** 38

**GLOUCS/77/2013**  **DV...L.T...PSR..N....S.HD..V...GS.VR.S** 38

**UK/58/2003**  **DV...L.T...PSR..N....S.HD..V...GS.VR.S** 38

**SUFF/91/1994**  **DV...L.T...PSR..N....S.HD..V...GS.VR.S** 38

**IRL/176/1994**  **DV...L.T...PSR..N....S.HD..V...GS.VR.S** 38

**IRL/966/2009**  **DV...L.T...PSR..N....S.HD..V...GS.VR.S** 38

**IRL/968/2009**  **DV...L.T...PSR..N....S.HD..V...GS.VR.S** 38

**IRL/778/1998**  **DV...L.T...PSR..N..N.S.HD..V...GS.VR.S** 38

**IRL/081/1998**  **DV...L.T...PSR..N..N.S.HD..V...GS.VR.S** 38

**IRL/942/2005**  **DV...L.T...PSR..N..N.S.HD..V...GS.VR.S** 38

**IRL/853/2008**  **DV...L.T...PSR..N..N.S.HD..V...GS.VR.S** 38

**IRL/907/2008**  **DV...L.T...PSR..N..N.S.HD..V...GS.VR.S** 38

**IRL/585/2008**  **DV...L.T...PSR..N..N.S.HD..V...GS.VR.S** 38

**IRL/849/2008**  **DV...L.T...PSR..N..N.S.HD..V...GS.VR.S** 38

**IRL/470/2008**  **DV...L.T...PSR..N..N.S.HD..V...GS.VR.S** 38

**IRL/471/2008**  **DV...L.T...PSR..N..N.S.HD..V...GS.VR.S** 38

**IRL/082/2011**  **DV...L.T...PSR..N..N.S.HD..V...GS.VR.S** 38

**IRL/684/2012**  **DV...L.T...PSR..N..N.S.HD..V...GS.VR.S** 38

**IRL/705/2015**  **DV...L.T...PSR..N..N.S.HD..V...GS.VR.S** 38

**IRL/286/2015**  **DV...L.T...PSR..N..N.S.HD..V...GS.VR.S** 38

**IRL/776/2015**  **DV...L.T...PSR..N..N.S.HD..V...GS.VR.S** 38

**IRL/307/2015**  **DV...L.T...PSR..N..N.S.HD..V...GS.VR.S** 38

**IRL/035/2016**  **DV...L.T...PSR..N..N.S.HD..V...GS.VR.S** 38

**IRL/160/2016**  **DV...L.T...PSR..N..N.S.HD..V...GS.VR.S** 38

**IRL/806/2001**  **DV...L.T.I.PSR..N..N.S.HD..V...GS.VR.S** 38

**IRL/609/2013**  **DV...L.T.I.PSR..N..N.S.HD..V...GS.VR.S** 38

**IRL/618/1992**  **DV...L.T.I.PSR..N..N.S.HD..V...GS.VR.S** 38

**IRL/608/1993**  **DV...L.T.I.PSR..N..N.S.HD..V...GS.VR.S** 38

**IRL/253/1994**  **DV...L.T.I.PSR..N..N.S.HD..V...GS.VR.S** 38

**IRL/414/1998**  **DV...L.T.I.PSR..N..N.S.HD..V...GS.VR.S** 38

**IRL/597/1998**  **DV...L.T.I.PSR..N..N.S.HD..V...GS.VR.S** 38

**IRL/651/1999**  **DV...L.T.I.PSR..N..N.S.HD..V...GS.VR.S** 38

**IRL/660/2001**  **DV...L.T.I.PSR..N..N.S.HD..V...GS.VR.S** 38

**IRL/368/2002**  **DV...L.T.I.PSR..N..N.S.HD..V...GS.VR.S** 38

**Supplementary Figure S1 (Continued)**

**IRL/026/2004**  **DV...L.T.I.PSR..N..N.S.HD..V...GS.VR.S** 38

**IRL/187/2005**  **DV...L.T.I.PSR..N..N.S.HD..V...GS.VR.S** 38

**IRL/276/2005**  **DV...L.T.I.PSR..N..N.S.HD..V...GS.VR.S** 38

**IRL/228/2007**  **DV...L.T.I.PSR..N..N.S.HD..V...GS.VR.S** 38

**IRL/858/2008**  **DV...L.T.I.PSR..N..N.S.HD..V...GS.VR.S** 38

**IRL/557/2008**  **DV...L.T.I.PSR..N..N.S.HD..V...GS.VR.S** 38

**IRL/349/2010**  **DV...L.T.I.PSR..N..N.S.HD..V...GS.VR.S** 38

**IRL/697/2013**  **DV...L.T.I.PSR..N..N.S.HD..V...GS.VR.S** 38

**SUFF/87/2009**  **DV...L.T...PSR..N..NKS.HD..V...GS.VR.S** 38

**SUFF/82/2013**  **DV...L.T...PSR..N..NKS.HD..V...GS.VR.S** 38

**SUFF/89/2013**  **DV...L.T...PSR..N..NKS.HD..V...GS.VR.S** 38

**IRL/348/2002**  **DV...L.T...PSR..N..NKS.HD..V...GS.VR.S** 38

**IRL/070/2006**  **DV...L.T...PSR..N..NKS.HD..V...GS.VR.S** 38

**IRL/867/2006**  **DV...L.T...PSR..N..NKS.HD..V...GS.VR.S** 38

**IRL/295/2006**  **DV...L.T...PSR..N..NKS.HD..V...GS.VR.S** 38

**IRL/316/2009**  **DV...L.T...PSR..N..NKS.HD..V...GS.VR.S** 38

**IRL/574/2009**  **DV...L.T...PSR..N..NKS.HD..V...GS.VR.S** 38

**IRL/962/2009**  **DV...L.T...PSR..N..NKS.HD..V...GS.VR.S** 38

**IRL/043/2009**  **DV...L.T...PSR..N..NKS.HD..V...GS.VR.S** 38

**IRL/328/2009**  **DV...L.T...PSR..N..NKS.HD..V...GS.VR.S** 38

**IRL/394/2009**  **DV...L.T...PSR..N..NKS.HD..V...GS.VR.S** 38

**IRL/626/2009**  **DV...L.T...PSR..N..NKS.HD..V...GS.VR.S** 38

**IRL/774/2010**  **DV...L.T...PSR..N..NKS.HD..V...GS.VR.S** 38

**IRL/441/2010**  **DV...L.T...PSR..N..NKS.HD..V...GS.VR.S** 38

**IRL/678/2011**  **DV...L.T...PSR..N..NKS.HD..V...GS.VR.S** 38

**IRL/607/2013**  **DV...L.T...PSR..N..NKS.HD..V...GS.VR.S** 38

**IRL/372/2013**  **DV...L.T...PSR..N..NKS.HD..V...GS.VR.S** 38

**IRL/330/2016**  **DV...L.T...PSR..N..NKS.HD..V...GS.VR.S** 38

**IRL/242/2017**  **DV...L.T...PSR..N..NKS.HD..V...GS.VR.S** 38

**IRL/009/2017**  **DV...L.T...PSR..N..NKS.HD..V...GS.VR.S** 38

**BRISTOL/2/1993**  **DVNK.L.T...PSR..NPKNKSLHDGRVLHRGS.VRVS** 38

**EHV-1.V592**  **DVNK.L.T...PSR..NPKNKSLHDGRVLHRGSSVRVS** 38

**IRL/075/1990**  **DVNK.L.T...PSR..NPK.KSLHDGRVLHRGS.VRVS** 38

**IRL/069/1995**  **DVNK.L.T...PSR..NPKNKSLHDGRVLHRGS.VRVS** 38

**IRL/708/1992**  **DVNK.L.T...PSR..NPKNKSLHDGRVLHRGS.VRVS** 38

**IRL/255/1996**  **DVNK.L.T...PSR..NPKNKSLHDGRVLHRGS.VRVS** 38

**IRL/746/1997**  **DVNK.L.T...PSR..NPKNKSLHDGRVLHRGS.VRVS** 38

**IRL/928/1997**  **DVNK.L.T...PSR..NPKNKSLHDGRVLHRGS.VRVS** 38

**IRL/033/1999**  **DVNK.L.T...PSR..NPKNKSLHDGRVLHRGS.VRVS** 38

**IRL/511/2000**  **DVNK.L.T...PSR..NPKNKSLHDGRVLHRGS.VRVS** 38

**IRL/977/2004**  **DVNK.L.T...PSR..NPKNKSLHDGRVLHRGS.VRVS** 38

**IRL/451/2005**  **DVNK.L.T...PSR..NPKNKSLHDGRVLHRGS.VRVS** 38

**IRL/737/2005**  **DVNK.L.T...PSR..NPKNKSLHDGRVLHRGS.VRVS** 38

**IRL/117/2005**  **DVNK.L.T...PSR..NPKNKSLHDGRVLHRGS.VRVS** 38

**IRL/087/2006**  **DVNK.L.T...PSR..NPKNKSLHDGRVLHRGS.VRVS** 38

**IRL/580/2007**  **DVNK.L.T...PSR..NPKNKSLHDGRVLHRGS.VRVS** 38

**IRL/023/2008**  **DVNK.L.T...PSR..NPKNKSLHDGRVLHRGS.VRVS** 38

**IRL/388/2008**  **DVNK.L.T...PSR..NPKNKSLHDGRVLHRGS.VRVS** 38

**IRL/047/2008**  **DVNK.L.T...PSR..NPKNKSLHDGRVLHRGS.VRVS** 38

**IRL/351/2008**  **DVNK.L.T...PSR..NPKNKSLHDGRVLHRGS.VRVS** 38

**IRL/043/2008**  **DVNK.L.T...PSR..NPKNKSLHDGRVLHRGS.VRVS** 38

**IRL/267/2010**  **DVNK.L.T...PSR..NPKNKSLHDGRVLHRGS.VRVS** 38

**IRL/172/2012**  **DVNK.L.T...PSR..NPKNKSLHDGRVLHRGS.VRVS** 38

**IRL/920/2013**  **DVNK.L.T...PSR..NPKNKSLHDGRVLHRGS.VRVS** 38

**IRL/706/2015**  **DVNK.L.T...PSR..NPKNKSLHDGRVLHRGS.VRVS** 38

**IRL/658/2016**  **DVNK.L.T...PSR..NPKNKSLHDGRVLHRGS.VRVS** 38

**IRL/492/1994**  **DVNK.L.T...PSR..NPK.KSLHDGRV.HRGS.VRVS** 38

**Supplementary Figure S1**

Amino acid alignment of a 38aa artificial peptide sequence for 269 Irish and 3 Italian (total 372) EHV-1 isolates (see Supplementary Table S1) with 49 international EHV-1 isolates (see Supplementary Table S2) constructed using ClustalW. The concatenated amino acid sequence of each isolate was constructed using 31 amino acid differences between Ab4 and V592 and a further seven additional amino acid differences in ORFs 11 (R235M), 13 (A405T, E492K, T493I, A499T) and 14 (R628K, S692N). The accession codes for the 49 complete and partial EHV-1 genome sequences used in this alignment are detailed in Supplementary Table S2. Dots indicate amino acid identity and dashes indicate amino acid deletion. (---) represents deletion of three amino acids in ORF14.
