# Supplementary material for: Molecular Characterisation of Equine Herpesvirus 1 Isolates from Cases of Abortion, Respiratory and Neurological Disease in Ireland between 1990 and 2017
Source: Pathogens. 2019 Jan 15;8(1):7. doi: 10.3390/pathogens8010007 (PMC6471309; doi:10.3390/pathogens8010007)
Supplement: Supplementary file 1 [file pathogens-08-00007-s001.zip › SupplementaryData/Supplementary Table S1.docx]

Supplementary Table S1: Summary of EHV-1 strains genotyped and listed in order of U_L_ clades

| **No.** | **Outbreak** | **Premises** | **UL** | **ORF68** | **Strain** | **Date** | **ORF30** | **Disease expression** | **Practice** | **Sample** | **Passage** |
| --- | --- | --- | --- | --- | --- | --- | --- | --- | --- | --- | --- |
|  | **Number^1^** | **No.** | **Clade ^3^** | **Group ^4^** | **name** | **sampled** | **Marker^5^** |  | **No.** | **Type** | **Number** |
|  |  |  |  |  |  |  |  | **A/NFD=abortion and/or neonatal foal death;**  M=Multiple; N=Neurological; |  |  |  |
|  |  |  |  |  |  |  |  | **R=Respiratory; S=Single.** |  |  |  |
| 1 | 1 | 1+T1**^2^** | **1** | 2 | IRL/710/1990 | 22/01/1990 | **D752** | MA/NFD | PR25 | Multiple Tissues**^6^** | Yes, P1**^7^** |
| 2 | 2 | 1+T1**^2^** | **1** | 2 | IRL/164/1994 | 20/07/1994 | **D752** | MN | PR40 | Nasal swab | Yes, P1 |
| 3 | 3 | 22+T1 | **1** | 2 | IRL/325/1995 | 15/06/1995 | **D752** | SN | PR99 | Nasal swab | Yes, P1 |
| 4 | 4 | 26 | **1** | 2 | IRL/600/1997 | 09/01/997 | N752 | SA/NFD | PR38 | Multiple Tissues | No |
| 5 | 5 | 29 | **1** | 1 | IRL/497/1997 | 06/11/1997 | **D752** | MN | PR45 | Multiple Tissues | No |
| 6 | 5 | 29 | **1** | /**^8^** | IRL/584/1997 | 11/11/1997 | **D752** | MN | PR45 | Multiple Tissues | No |
| 7 | 6 | 78 | **1** | 2 | IRL/440/2005 | 04/10/2005 | **D752** | MN | PR14 | Nasal swab | Yes, P1 |
| 8 | 6 | 78 | **1** | / | IRL/443/2005 | 04/10/2005 | **D752** | MN | PR14 | Nasal swab | Yes, P1 |
| 9 | 7 | 114 | **1** | 2 | IRL/366/2016 | 09/12/2016 | N752 | SA/NFD | PR64 | Multiple Tissues | No |
| 10 | 8 | 116 | **1** | 2 | IRL/559/2009 | 27/02/2009 | N752 | SA/NFD | PR3 | Multiple Tissues | No |
| 11 | 9 | 133 | **1** | 2 | ITA/055/2011 | 17/01/2011 | N752 | MA/NFD | PR107 | Multiple Tissues | No |
| 12 | 9 | 133 | **1** | / | ITA/056/2011 | 17/01/2011 | N752 | MA/NFD | PR107 | Multiple Tissues | No |
| 13 | 10 | 173 | **1** | 2 | IRL/282/2015 | 23/03/2015 | **D752** | SA/NFD | PR34 | Multiple Tissues | No |
|  |  |  |  |  |  |  |  |  |  |  |  |
| 14 | 11 | 8 | **3** | 2 | IRL/962/1992 | 16/04/1992 | N752 | SA/NFD | PR20 | Multiple Tissues | Yes, P1 |
| 15 | 12 | 12 | **3** | / | IRL/154/1993 | 16/11/1993 | **D752** | SN | PR20 | Brain Tissue | Yes, P1 |
|  |  |  |  |  |  |  |  |  |  |  |  |
| 16 | 13 | 160 | **5** | 2 | IRL/916/2014 | 24/01/2014 | N752 | MA/NFD | PR51 | Multiple Tissues | No |
| 17 | 13 | 160 | **5** | / | IRL/546/2014 | 27/01/2014 | N752 | MA/NFD | PR51 | Multiple Tissues | No |
| 18 | 14 | 172 | **5** | 2 | IRL/801/2015 | 16/02/2015 | N752 | SA/NFD | PR82 | Multiple Tissues | No |
|  |  |  |  |  |  |  |  |  |  |  |  |
| 19 | 15 | 24 | **6** | 5 | IRL/919/2005 | 16/03/2005 | N752 | SA/NFD | PR72 | Multiple Tissues | No |
| 20 | 16 | 69 | **6** | 5 | IRL/025/2005 | 30/03/2005 | N752 | SA/NFD | PR7 | Multiple Tissues | No |
| 21 | 17 | 86 | **6** | 5 | IRL/130/2006 | 21/02/2006 | N752 | SA/NFD | PR53 | Multiple Tissues | No |
| 22 | 18 | 107 | **6** | 5 | IRL/177/2008 | 18/03/2008 | N752 | SA/NFD | PR70 | Multiple Tissues | No |
| 23 | 19 | 112 | **6** | 5 | IRL/307/2008 | 23/05/2008 | N752 | SA/NFD | PR86 | Multiple Tissues | No |
| 24 | 20 | 113 | **6** | 5 | IRL/238/2008 | 15/06/2008 | N752 | SA/NFD | PR86 | Multiple Tissues | No |
| 25 | 21 | 114+T5 | **6** | 5 | IRL/766/2008 | 05/09/2008 | N752 | SN | PR64 | Nasal swab | Yes, P1 |
| 26 | 21 | 114+T5 | **6** | / | IRL/155/2008 | 20/11/2008 | N752 | SA/NFD | PR64 | Multiple Tissues | No |
| 27 | 22 | 120 | **6** | 5 | IRL/671/2009 | 25/04/2009 | N752 | SA/NFD | PR73 | Multiple Tissues | No |
| 28 | 23 | 132 | **6** | 5 | IRL/721/2010 | 08/10/2010 | N752 | SA/NFD | PR86 | Multiple Tissues | No |
| 29 | 24 | 146 | **6** | 5 | IRL/169/2012 | 25/02/2012 | N752 | SA/NFD | PR102 | Multiple Tissues | No |
| 30 | 25 | 148 | **6** | 5 | IRL/570/2012 | 05/03/2012 | N752 | MA/NFD | PR3 | Multiple Tissues | No |
| 31 | 25 | 148 | **6** | / | IRL/572/2012 | 05/03/2012 | N752 | MA/NFD | PR3 | Multiple Tissues | No |
| 32 | 25 | 148 | **6** | / | IRL/863/2012 | 12/03/2012 | N752 | MA/NFD | PR3 | Multiple Tissues | No |
| 33 | 25 | 148 | **6** | / | IRL/948/2012 | 13/03/2012 | N752 | MA/NFD | PR3 | Multiple Tissues | No |
| 34 | 26 | 161 | **6** | 5 | IRL/159/2014 | 27/01/2014 | N752 | SA/NFD | PR75 | Multiple Tissues | No |
| 35 | 27 | 167 | **6** | / | IRL/031/2014 | 05/11/2014 | N752 | SA/NFD | PR65 | Multiple Tissues | No |
| 36 | 28 | 179 | **6** | 5 | IRL/983/2015 | 20/10/2015 | N752 | SA/NFD | PR51 | Multiple Tissues | No |
| 37 | 29 | 182 | **6** | 5 | IRL/050/2015 | 09/12/2015 | N752 | SA/NFD | PR16 | Multiple Tissues | No |
| 38 | 30 | 184 | **6** | 5 | IRL/784/2016 | 13/02/2016 | N752 | SA/NFD | PR69 | Multiple Tissues | No |
| 39 | 31 | 186 | **6** | 5 | IRL/704/2016 | 03/03/2016 | N752 | SA/NFD | PR33 | Multiple Tissues | No |
| 40 | 32 | 198 | **6** | 5 | IRL/425/2017 | 01/02/2017 | N752 | SA/NFD | PR47 | Multiple Tissues | No |
| 41 | 33 | 199 | **6** | 5 | IRL/426/2017 | 11/02/2017 | N752 | SA/NFD | PR8 | Multiple Tissues | No |
| 42 | 34 | 209 | **6** | 5 | IRL/001/2017 | 07/07/2017 | N752 | R | PR75 | Nasal swab | No |
| 43 | 35 | 214 | **6** | 5 | IRL/837/2007 | 31/01/2007 | N752 | SA/NFD | PR12 | Multiple Tissues | No |
|  |  |  |  |  |  |  |  |  |  |  |  |
| 44 | 36 | 4 | **7** | 3 | IRL/905/1991 | 04/02/1991 | N752 | MA/NFD | PR39 | Nasal swab | Yes, P1 |
| 45 | 37 | 5 | **7** | 3 | IRL/001/1991 | 17/04/1991 | N752 | SA/NFD | PR47 | Multiple Tissues | No |
| 46 | 38 | 6 | **7** | 3 | IRL/017/1992 | 04/03/1992 | N752 | SA/NFD | PR19 | Multiple Tissues | Yes, P1 |
| 47 | 39 | 10 | **7** | 3 | IRL/212/1993 | 02/03/1993 | N752 | SA/NFD | PR35 | Multiple Tissues | No |
| 48 | 40 | 11 | **7** | 3 | IRL/212/1999 | 04/06/1999 | N752 | SN | PR10 | Nasal swab | Yes, P1 |
| 49 | 41 | 13 | **7** | 3 | IRL/064/1994 | 16/01/1994 | N752 | SA/NFD | PR88 | Multiple Tissues | No |
| 50 | 42 | 16+T2 | **7** | 3 | IRL/206/1994 | 16/05/1994 | N752 | SN | PR11 | Nasal swab | Yes, P1 |
| 51 | 43 | 17 | **7** | / | IRL/048/1994 | 08/06/1994 | **D752** | SN | PR44 | Multiple Tissues | Yes, P1 |
| 52 | 44 | 19 | **7** | 3 | IRL/609/1995 | 07/02/1995 | N752 | SA/NFD | PR105 | Multiple Tissues | No |
| 53 | 45 | 21 | **7** | 3 | IRL/776/1995 | 04/05/1995 | N752 | SA/NFD | PR104 | Multiple Tissues | No |
| 54 | 46 | 24 | **7** | 3 | IRL/096/1996 | 15/04/1996 | N752 | SA/NFD | PR41 | Multiple Tissues | No |
| 55 | 47 | 28 | **7** | 3 | IRL/197/2017 | 28/02/2017 | N752 | SA/NFD | PR34 | Multiple Tissues | No |
| 56 | 48 | 34 | **7** | 3 | IRL/463/1999 | 08/04/1999 | N752 | SA/NFD | PR28 | Multiple Tissues | No |
| 57 | 49 | 36 | **7** | 3 | IRL/045/1999 | 12/11/1999 | N752 | SA/NFD | PR88 | Multiple Tissues | No |
| 58 | 50 | 38 | **7** | 3 | IRL/775/2000 | 11/05/2000 | N752 | SA/NFD | PR88 | Multiple Tissues | No |
| 59 | 51 | 39 | **7** | 3 | IRL/159/2000 | 08/06/2000 | N752 | SA/NFD | PR91 | Multiple Tissues | No |
| 60 | 52 | 40 | **7** | / | IRL/441/2000 | 05/07/2000 | **D752** | SN | PR34 | Multiple Tissues | Yes, P1 |
| 61 | 53 | 42 | **7** | 3 | IRL/074/2001 | 27/03/2001 | N752 | MA/NFD | PR59 | Multiple Tissues | No |
| 62 | 54 | 46 | **7** | 3 | IRL/009/2002 | 18/02/2002 | N752 | SA/NFD | PR38 | Multiple Tissues | No |
| 63 | 55 | 48 | **7** | 3 | IRL/461/2002 | 05/04/2002 | **D752** | MN | PR54 | Nasal swab | No |
| 64 | 56 | 49 | **7** | 3 | IRL/813/2003 | 13/02/2003 | N752 | SA/NFD | PR34 | Multiple Tissues | No |
| 65 | 57 | 51 | **7** | 3 | IRL/311/2003 | 07/04/2003 | N752 | SA/NFD | PR87 | Multiple Tissues | No |
| 66 | 58 | 52 | **7** | 3 | IRL/650/2004 | 15/01/2004 | N752 | SA/NFD | PR34 | Multiple Tissues | No |
| 67 | 59 | 55 | **7** | 3 | IRL/942/2004 | 13/09/2004 | N752 | SN | PR1 | Multiple Tissues | No |
| 68 | 60 | 56 | **7** | 3 | IRL/882/2004 | 20/12/2004 | N752 | SA/NFD | PR62 | Multiple Tissues | No |
| 69 | 61 | 58 | **7** | 3 | IRL/094/2005 | 25/02/2005 | N752 | SA/NFD | PR7 | Multiple Tissues | No |
| 70 | 62 | 60 | **7** | 3 | IRL/992/2005 | 08/03/2005 | N752 | MA/NFD | PR103 | Multiple Tissues | No |
| 71 | 63 | 61 | **7** | 3 | IRL/541/2005 | 12/03/2005 | N752 | SA/NFD | PR34 | Multiple Tissues | No |
| 72 | 64 | 64 | **7** | 3 | IRL/279/2005 | 22/03/2005 | N752 | MA/NFD | PR66 | Multiple Tissues | No |
| 73 | 65 | 65 | **7** | 3 | IRL/694/2005 | 24/03/2005 | N752 | SA/NFD | PR3 | Nasal swab | Yes, P1 |
| 74 | 66 | 66 | **7** | 3 | IRL/926/2005 | 27/03/2005 | N752 | SA/NFD | PR15 | Multiple Tissues | No |
| 75 | 67 | 67 | **7** | 3 | IRL/024/2005 | 29/03/2005 | N752 | SA/NFD | PR111 | Multiple Tissues | No |
| 76 | 68 | 68 | **7** | 3 | IRL/998/2005 | 29/03/2005 | N752 | SA/NFD | PR110 | Multiple Tissues | No |
| 77 | 69 | 73 | **7** | 3 | IRL/065/2005 | 16/04/2005 | N752 | SA/NFD | PR34 | Multiple Tissues | No |
| 78 | 70 | 74 | **7** | 3 | IRL/071/2005 | 17/04/2005 | N752 | SA/NFD | PR7 | Multiple Tissues | No |
| 79 | 71 | 74 | **7** | / | IRL/433/2014 | 28/01/2014 | N752 | MA/NFD | PR7 | Multiple Tissues | No |
| 80 | 71 | 74 | **7** | 3 | IRL/749/2014 | 08/03/2014 | N752 | MA/NFD | PR7 | Multiple Tissues | No |
| 81 | 72 | 75 | **7** | 3 | IRL/073/2005 | 17/04/2005 | N752 | SA/NFD | PR7 | Multiple Tissues | No |
| 82 | 73 | 76 | **7** | 3 | IRL/125/2005 | 18/04/2005 | N752 | SA/NFD | PR34 | Multiple Tissues | No |
| 83 | 74 | 79 | **7** | 3 | IRL/952/2005 | 15/11/2005 | N752 | SA/NFD | PR13 | Multiple Tissues | No |
| 84 | 75 | 80 | **7** | 3 | IRL/055/2005 | 21/11/2005 | N752 | SA/NFD | PR24 | Multiple Tissues | No |
| 85 | 76 | 81 | **7** | 3 | IRL/054/2005 | 21/11/2005 | N752 | SA/NFD | PR85 | Multiple Tissues | No |
| 86 | 77 | 82 | **7** | 3 | IRL/108/2005 | 24/12/2005 | N752 | SA/NFD | PR34 | Multiple Tissues | No |
| 87 | 78 | 83 | **7** | 3 | IRL/124/2005 | 30/12/2005 | N752 | SA/NFD | PR47 | Multiple Tissues | No |
| 88 | 79 | 84 | **7** | 3 | IRL/943/2006 | 10/01/2006 | N752 | SA/NFD | PR50 | Multiple Tissues | No |
| 89 | 80 | 85 | **7** | / | IRL/820/2006 | 16/01/2006 | N752 | MA/NFD | PR21 | Multiple Tissues | No |
| 90 | 80 | 85 | **7** | 3 | IRL/315/2006 | 03/03/2006 | N752 | MA/NFD | PR21 | Multiple Tissues | No |
| 91 | 80 | 85 | **7** | / | IRL/374/2006 | 04/03/2006 | N752 | MA/NFD | PR21 | Multiple Tissues | No |
| 92 | 80 | 85 | **7** | / | IRL/935/2006 | 10/03/2006 | N752 | MA/NFD | PR21 | Multiple Tissues | No |
| 93 | 81 | 87 | **7** | 3 | IRL/695/2006 | 23/02/2006 | N752 | SA/NFD | PR60 | Multiple Tissues | No |
| 94 | 82 | 88 | **7** | 3 | IRL/754/2006 | 27/02/2006 | N752 | SA/NFD | PR98 | Multiple Tissues | No |
| 95 | 83 | 89 | **7** | 3 | IRL/596/2017 | 16/02/2017 | N752 | SA/NFD | PR4 | Multiple Tissues | No |
| 96 | 84 | 90 | **7** | 3 | IRL/558/2006 | 07/03/2006 | N752 | SA/NFD | PR60 | Multiple Tissues | No |
| 97 | 85 | 92 | **7** | / | IRL/824/2007 | 05/01/2007 | N752 | MA/NFD | PR100 | Multiple Tissues | No |
| 98 | 85 | 92 | **7** | 3 | IRL/825/2007 | 05/01/2007 | N752 | MA/NFD | PR10 | Multiple Tissues | No |
| 99 | 86 | 93 | **7** | 3 | IRL/539/2006 | 18/03/2006 | N752 | SA/NFD | PR61 | Multiple Tissues | No |
| 100 | 87 | 94 | **7** | 3 | IRL/123/2006 | 24/03/2006 | N752 | SA/NFD | PR39 | Multiple Tissues | No |
| 101 | 88 | 95 | **7** | 3 | IRL/898/2007 | 08/03/2007 | N752 | SA/NFD | PR34 | Multiple Tissues | No |
| 102 | 89 | 96 | **7** | 3 | IRL/670/2007 | 19/03/2007 | N752 | MA/NFD | PR39 | Multiple Tissues | No |
| 103 | 89 | 96 | **7** | / | IRL/671/2007 | 19/03/2007 | N752 | MA/NFD | PR39 | Multiple Tissues | No |
| 104 | 90 | 97 | **7** | 3 | IRL/895/2007 | 31/03/2007 | N752 | SA/NFD | PR34 | Multiple Tissues | No |
| 105 | 91 | 101 | **7** | 3 | IRL/410/2008 | 04/02/2008 | N752 | SA/NFD | PR26 | Multiple Tissues | No |
| 106 | 92 | 103 | **7** | 3 | IRL/060/2008 | 08/02/2008 | N752 | SA/NFD | PR3 | Multiple Tissues | No |
| 107 | 93 | 107 | **7** | 3 | IRL/040/2016 | 20/12/2016 | N752 | SA/NFD | PR70 | Multiple Tissues | No |
| 108 | 94 | 109 | **7** | 3 | IRL/368/2008 | 15/04/2008 | N752 | MA/NFD | PR21 | Multiple Tissues | No |
| 109 | 95 | 111 | **7** | 3 | IRL/569/2008 | 13/05/2008 | N752 | SA/NFD | PR3 | Multiple Tissues | No |
| 110 | 96 | 123 | **7** | 3 | IRL/313/2010 | 15/02/2010 | N752 | MA/NFD | PR86 | Multiple Tissues | No |
| 111 | 96 | 123 | **7** | / | IRL/851/2010 | 21/02/2010 | N752 | MA/NFD | PR86 | Multiple Tissues | No |
| 112 | 96 | 123 | **7** | / | IRL/130/2010 | 26/02/2010 | N752 | MA/NFD | PR86 | Multiple Tissues | No |
| 113 | 96 | 123 | **7** | / | IRL/331/2010 | 02/03/2010 | N752 | MA/NFD | PR86 | Multiple Tissues | No |
| 114 | 97 | 123 | **7** | / | IRL/031/2012 | 07/11/2012 | N752 | MA/NFD | PR86 | Multiple Tissues | No |
| 115 | 98 | 124 | **7** | 3 | IRL/534/2010 | 05/03/2010 | N752 | SA/NFD | PR78 | Multiple Tissues | No |
| 116 | 99 | 127 | **7** | 3 | IRL/631/2010 | 25/03/2010 | N752 | SA/NFD | PR88 | Multiple Tissues | No |
| 117 | 100 | 129 | **7** | 3 | IRL/481/2010 | 21/04/2010 | N752 | SA/NFD | PR80 | Multiple Tissues | No |
| 118 | 101 | 130 | **7** | 3 | IRL/467/2010 | 11/05/2010 | N752 | SA/NFD | PR88 | Multiple Tissues | No |
| 119 | 102 | 131 | **7** | 3 | IRL/114/2010 | 19/07/2010 | N752 | SA/NFD | PR34 | Multiple Tissues | No |
| 120 | 103 | 134 | **7** | 3 | IRL/923/2011 | 25/01/2011 | N752 | SA/NFD | PR113 | Multiple Tissues | No |
| 121 | 104 | 136 | **7** | 3 | IRL/795/2011 | 13/04/2011 | N752 | SA/NFD | PR77 | Multiple Tissues | No |
| 122 | 105 | 138 | **7** | 3 | IRL/409/2011 | 28/04/2011 | N752 | MA/NFD | PR47 | Multiple Tissues | No |
| 123 | 106 | 139 | **7** | 3 | IRL/745/2011 | 10/05/2011 | N752 | MA/NFD | PR3 | Multiple Tissues | No |
| 124 | 107 | 140 | **7** | / | IRL/902/2011 | 17/05/2011 | N752 | MA/NFD | PR72 | Multiple Tissues | No |
| 125 | 108 | 144 | **7** | 3 | IRL/013/2012 | 03/01/2012 | N752 | SA/NFD | PR29 | Multiple Tissues | No |
| 126 | 109 | 147 | **7** | 3 | IRL/241/2012 | 27/02/2012 | N752 | SA/NFD | PR72 | Multiple Tissues | No |
| 127 | 110 | 148 | **7** | 3 | IRL/001/2015 | 01/01/2015 | N752 | SA/NFD | PR3 | Multiple Tissues | No |
| 128 | 111 | 150 | **7** | 3 | IRL/894/2013 | 04/02/2013 | N752 | MA/NFD | PR77 | Multiple Tissues | No |
| 129 | 111 | 150 | **7** | / | IRL/249/2013 | 20/02/2013 | N752 | MA/NFD | PR3 | Multiple Tissues | No |
| 130 | 112 | 156 | **7** | / | IRL/222/2013 | 18/04/2013 | N752 | MA/NFD | PR86 | Multiple Tissues | No |
| 131 | 112 | 156 | **7** | 3 | IRL/223/2013 | 18/04/2013 | N752 | MA/NFD | PR86 | Multiple Tissues | No |
| 132 | 113 | 158 | **7** | 3 | IRL/332/2013 | 22/04/2013 | N752 | SA/NFD | PR34 | Multiple Tissues | No |
| 133 | 114 | 159 | **7** | 3 | IRL/559/2006 | 07/03/2006 | N752 | MA/NFD | PR93 | Multiple Tissues | No |
| 134 | 114 | 159 | **7** | / | IRL/396/2006 | 28/03/2006 | N752 | MA/NFD | PR34 | Multiple Tissues | No |
| 135 | 115 | 160 | **7** | 3 | IRL/739/2015 | 03/04/2015 | N752 | SA/NFD | PR110 | Multiple Tissues | No |
| 136 | 116 | 163 | **7** | 3 | IRL/694/2014 | 06/03/2014 | N752 | SA/NFD | PR110 | Multiple Tissues | No |
| 137 | 117 | 164 | **7** | 3 | IRL/052/2014 | 12/04/2014 | N752 | SA/NFD | PR7 | Multiple Tissues | No |
| 138 | 118 | 165 | **7** | 3 | IRL/967/2014 | 11/05/2014 | N752 | SA/NFD | PR9 | Multiple Tissues | No |
| 139 | 119 | 166 | **7** | 3 | IRL/333/2014 | 27/05/2014 | N752 | SA/NFD | PR34 | Multiple Tissues | No |
| 140 | 120 | 169 | **7** | 3 | IRL/878/2015 | 21/01/2015 | N752 | SA/NFD | PR58 | Multiple Tissues | No |
| 141 | 121 | 175 | **7** | 3 | IRL/550/2015 | 30/03/2015 | N752 | SA/NFD | PR83 | Multiple Tissues | No |
| 142 | 122 | 176 | **7** | 3 | IRL/904/2015 | 09/04/2015 | N752 | SA/NFD | PR83 | Multiple Tissues | No |
| 143 | 123 | 177 | **7** | 3 | IRL/987/2015 | 13/04/2015 | N752 | MA/NFD | PR66 | Multiple Tissues | No |
| 144 | 124 | 178 | **7** | 3 | IRL/564/2015 | 27/04/2015 | N752 | SA/NFD | PR3 | Multiple Tissues | No |
| 145 | 125 | 180 | **7** | 3 | IRL/596/2015 | 10/11/2015 | N752 | SA/NFD | PR29 | Multiple Tissues | No |
| 146 | 126 | 183 | **7** | 3 | IRL/617/2016 | 18/01/2016 | N752 | SA/NFD | PR67 | Multiple Tissues | No |
| 147 | 127 | 185 | **7** | 3 | IRL/675/2016 | 02/03/2016 | N752 | MA/NFD | PR68 | Multiple Tissues | No |
| 148 | 128 | 190 | **7** | 3 | IRL/991/2016 | 07/04/2016 | **D752** | SA/NFD | PR66 | Multiple Tissues | No |
| 149 | 129 | 191 | **7** | 3 | IRL/061/2016 | 08/04/2016 | N752 | SA/NFD | PR3 | Multiple Tissues | No |
| 150 | 130 | 192 | **7** | 3 | IRL/067/2016 | 11/04/2016 | N752 | SA/NFD | PR68 | Multiple Tissues | No |
| 151 | 131 | 193 | **7** | 3 | IRL/280/2016 | 21/04/2016 | N752 | SA/NFD | PR34 | Multiple Tissues | No |
| 152 | 132 | 200 | **7** | 3 | IRL/467/2017 | 06/03/2017 | N752 | SA/NFD | PR86 | Multiple Tissues | No |
| 153 | 133 | 201 | **7** | 3 | IRL/573/2017 | 28/03/2017 | **D752** | SA/NFD | PR34 | Multiple Tissues | No |
| 154 | 134 | 202 | **7** | 3 | IRL/948/2017 | 06/04/2017 | N752 | SA/NFD | PR79 | Multiple Tissues | No |
| 155 | 135 | 203 | **7** | 3 | IRL/332/2017 | 14/04/2017 | N752 | SA/NFD | PR66 | Multiple Tissues | No |
| 156 | 136 | 204 | **7** | 3 | IRL/356/2017 | 15/04/2017 | N752 | SA/NFD | PR58 | Multiple Tissues | No |
| 157 | 137 | 205 | **7** | 3 | IRL/726/2017 | 25/04/2017 | N752 | SA/NFD | PR95 | Multiple Tissues | No |
| 158 | 138 | 207 | **7** | 3 | IRL/145/2017 | 04/05/2017 | N752 | R | PR58 | Nasal swab | No |
| 159 | 139 | 208 | **7** | 3 | IRL/024/2017 | 25/05/2017 | N752 | SA/NFD | PR34 | Multiple Tissues | No |
| 160 | 140 | 215 | **7** | 3 | IRL/979/2007 | 03/03/2007 | N752 | SA/NFD | PR7 | Multiple Tissues | No |
| 161 | 141 | 216 | **7** | 3 | IRL/533/2006 | 17/03/2006 | N752 | SA/NFD | PR85 | Multiple Tissues | No |
|  |  |  |  |  |  |  |  |  |  |  |  |
| 162 | 142 | 3 | **8** | 4 | IRL/542/1990 | 06/05/1990 | **D752** | MN | PR48 | Brain Tissue | Yes, P1 |
| 163 | 143 | 11 | **8** | 4 | IRL/268/2001 | 19/11/2001 | **D752** | SA/NFD | PR55 | Multiple Tissues | No |
| 164 | 144 | 28 | **8** | 4 | IRL/961/2007 | 30/01/2007 | **D752** | MA/NFD | PR34 | Multiple Tissues | No |
| 165 | 144 | 28 | **8** | / | IRL/568/2007 | 20/02/2007 | **D752** | MA/NFD | PR34 | Multiple Tissues | No |
| 166 | 145 | 43 | **8** | 4 | IRL/634/2001 | 19/04/2001 | **D752** | SA/NFD | PR12 | Multiple Tissues | Yes, P1 |
| 167 | 146 | 45 | **8** | 4 | IRL/612/2010 | 17/11/2010 | **D752** | SA/NFD | PR6 | Multiple Tissues | No |
| 168 | 147 | 50 | **8** | / | ITA/944/2003 | 03/03/2003 | **D752** | MA/NFD | PR8 | Multiple Tissues | No |
| 169 | 148 | 89 | **8** | 4 | IRL/184/2006 | 17/10/2006 | **D752** | SA/NFD | PR15 | Multiple Tissues | No |
| 170 | 149 | 114 | **8** | 4 | IRL/439/2015 | 21/09/2015 | **D752** | SA/NFD | PR64 | Multiple Tissues | No |
| 171 | 150 | 115 | **8** | 4 | IRL/995/2008 | 29/10/2008 | **D752** | SA/NFD | PR89 | Multiple Tissues | No |
| 172 | 151 | 162 | **8** | 4 | IRL/218/2014 | 05/02/2014 | **D752** | SA/NFD | PR34 | Multiple Tissues | No |
| 173 | 152 | 194 | **8** | 4 | IRL/917/2016 | 29/04/2016 | **D752** | MN | PR95 | Multiple Tissues | No |
| 174 | 153 | 210 | **8** | / | IRL/642/2007 | 22/01/2007 | **D752** | MA/NFD | PR73 | Multiple Tissues | No |
| 175 | 154 | 213 | **8** | 4 | IRL/306/2006 | 14/11/2006 | **D752** | MA/NFD | PR72 | Multiple Tissues | No |
|  |  |  |  |  |  |  |  |  |  |  |  |
| 176 | 155 | 2 | **9** | 6 | IRL/075/1990 | 09/03/1990 | **D752** | SA/NFD | PR88 | Multiple Tissues | No |
| 177 | 156 | 9 | **9** | 6 | IRL/708/1992 | 19/05/1992 | N752 | SA/NFD | PR46 | Multiple Tissues | No |
| 178 | 157 | 18 | **9** | 6 | IRL/492/1994 | 29/06/1994 | **D752** | MN | PR88 | Multiple Tissues | No |
| 179 | 158 | 20 | **9** | 6 | IRL/069/1995 | 25/03/1995 | N752 | SA/NFD | PR88 | Multiple Tissues | No |
| 180 | 159 | 25 | **9** | 6 | IRL/255/1996 | 21/04/1996 | N752 | SA/NFD | PR105 | Multiple Tissues | No |
| 181 | 160 | 27 | **9** | 6 | IRL/746/1997 | 27/01/1997 | N752 | SA/NFD | PR5 | Nasal swab | Yes, P1 |
| 182 | 161 | 28 | **9** | 6 | IRL/928/1997 | 30/01/1997 | N752 | SA/NFD |  | Multiple Tissues | No |
| 183 | 162 | 33 | **9** | 6 | IRL/033/1999 | 18/03/1999 | N752 | MA/NFD | PR90 | Multiple Tissues | No |
| 184 | 163 | 37 | **9** | 6 | IRL/511/2000 | 08/02/2000 | N752 | SA/NFD | PR25 | Multiple Tissues | Yes, P1 |
| 185 | 164 | 53 | **9** | 6 | IRL/977/2004 | 26/04/2004 | N752 | SA/NFD | PR58 | Multiple Tissues | No |
| 186 | 165 | 59 | **9** | 6 | IRL/451/2005 | 01/03/2005 | N752 | MA/NFD | PR81 | Multiple Tissues | No |
| 187 | 166 | 71 | **9** | 6 | IRL/737/2005 | 05/04/2005 | N752 | SA/NFD | PR88 | Multiple Tissues | No |
| 188 | 167 | 77 | **9** | 6 | IRL/117/2005 | 10/05/2005 | N752 | SA/NFD | PR83 | Multiple Tissues | No |
| 189 | 168 | 99 | **9** | 6 | IRL/580/2007 | 24/04/2007 | N752 | SA/NFD | PR22 | Multiple Tissues | No |
| 190 | 169 | 105 | **9** | 6 | IRL/023/2008 | 05/03/2008 | N752 | MA/NFD | PR110 | Multiple Tissues | No |
| 191 | 169 | 105 | **9** | / | IRL/047/2008 | 14/03/2008 | N752 | MA/NFD | PR46 | Multiple Tissues | No |
| 192 | 169 | 105 | **9** | / | IRL/351/2008 | 19/03/2008 | N752 | MA/NFD | PR110 | Multiple Tissues | No |
| 193 | 169 | 105 | **9** | / | IRL/043/2008 | 26/03/2008 | N752 | MA/NFD | PR110 | Multiple Tissues | No |
| 194 | 170 | 106 | **9** | 6 | IRL/388/2008 | 08/03/2008 | N752 | SA/NFD | PR69 | Multiple Tissues | No |
| 195 | 171 | 125 | **9** | 6 | IRL/267/2010 | 19/03/2010 | N752 | SA/NFD | PR26 | Multiple Tissues | No |
| 196 | 172 | 145 | **9** | 6 | IRL/172/2012 | 04/01/2012 | N752 | MA/NFD | PR47 | Multiple Tissues | No |
| 197 | 173 | 155 | **9** | 6 | IRL/920/2013 | 11/04/2013 | N752 | SA/NFD | PR88 | Multiple Tissues | No |
| 198 | 174 | 171 | **9** | 6 | IRL/706/2015 | 12/02/2015 | N752 | MA/NFD | PR85 | Multiple Tissues | No |
| 199 | 175 | 189 | **9** | 6 | IRL/658/2016 | 30/03/2016 | N752 | SA/NFD | PR66 | Multiple Tissues | No |
| 200 | 176 | 212 | **9** | 6 | IRL/087/2006 | 13/03/2006 | N752 | SA/NFD | PR92 | Multiple Tissues | No |
|  |  |  |  |  |  |  |  |  |  |  |  |
| 201 | 177 | 31 | **10** | 4 | IRL/774/2010 | 27/03/2010 | N752 | SA/NFD | PR110 | Multiple Tissues | No |
| 202 | 178 | 45 | **10** | 4 | IRL/348/2002 | 07/02/2002 | N752 | SA/NFD | PR52 | Multiple Tissues | No |
| 203 | 179 | 45 | **10** | 4 | IRL/867/2006 | 06/05/2006 | N752 | MA/NFD | PR75 | Multiple Tissues | No |
| 204 | 180 | 91 | **10** | 4 | IRL/295/2006 | 29/12/2006 | N752 | SA/NFD | PR32 | Multiple Tissues | No |
| 205 | 181 | 117 | **10** | 4 | IRL/316/2009 | 30/03/2009 | N752 | MA/NFD | PR72 | Multiple Tissues | No |
| 206 | 181 | 117 | **10** | / | IRL/574/2009 | 03/04/2009 | N752 | MA/NFD | PR72 | Nasal swab | No |
| 207 | 181 | 117 | **10** | / | IRL/962/2009 | 10/04/2009 | N752 | MA/NFD | PR72 | Nasal swab | No |
| 208 | 181 | 117 | **10** | / | IRL/043/2009 | 14/04/2009 | N752 | MA/NFD | PR72 | Nasal swab | No |
| 209 | 182 | 119 | **10** | 4 | IRL/328/2009 | 19/04/2009 | N752 | SA/NFD | PR18 | Multiple Tissues | No |
| 210 | 183 | 121+T4 | **10** | 4 | IRL/394/2009 | 11/05/2009 | N752 | SN | PR34 | Nasal swab | Yes, P1 |
| 211 | 184 | 122+T4 | **10** | 4 | IRL/626/2009 | 16/05/2009 | N752 | SA/NFD | PR17 | Nasal swab | Yes, P1 |
| 212 | 185 | 128 | **10** | 4 | IRL/441/2010 | 07/04/2010 | N752 | SA/NFD | PR3 | Multiple Tissues | No |
| 213 | 186 | 143 | **10** | 4 | IRL/678/2011 | 27/09/2011 | N752 | SA/NFD | PR71 | Multiple Tissues | No |
| 214 | 187 | 151 | **10** | 4 | IRL/607/2013 | 25/02/2013 | N752 | SA/NFD | PR71 | Multiple Tissues | No |
| 215 | 188 | 153 | **10** | 4 | IRL/372/2013 | 11/03/2013 | N752 | MA/NFD | PR71 | Multiple Tissues | No |
| 216 | 189 | 196 | **10** | 4 | IRL/330/2016 | 20/12/2016 | N752 | SA/NFD | PR55 | Multiple Tissues | No |
| 217 | 190 | 197 | **10** | 4 | IRL/242/2017 | 31/01/2017 | N752 | SA/NFD | PR34 | Multiple Tissues | No |
| 218 | 191 | 206 | **10** | 4 | IRL/009/2017 | 02/05/2017 | N752 | SA/NFD | PR6 | Multiple Tissues | No |
| 219 | 192 | 220 | **10** | 4 | IRL/070/2006 | 29/04/2006 | N752 | SA/NFD | PR3 | Multiple Tissues | No |
|  |  |  |  |  |  |  |  |  |  |  |  |
| 220 | 193 | 15+T2 | **11** | / | IRL/176/1994 | 16/05/1994 | **D752** | MN | PR42 | Nasal swab | Yes, P1 |
| 221 | 194 | 31 | **11** | / | IRL/778/1998 | 26/04/1998 | N752 | SA/NFD | PR51 | Multiple Tissues | No |
| 222 | 195 | 32 | **11** | / | IRL/081/1998 | 16/10/1998 | N752 | SA/NFD | PR43 | Multiple Tissues | No |
| 223 | 196 | 72 | **11** | 3 | IRL/942/2005 | 14/04/2005 | N752 | MA/NFD | PR31 | Multiple Tissues | No |
| 224 | 197 | 100 | **11** | 3 | IRL/853/2008 | 22/12/2007 | N752 | MA/NFD | PR77 | Multiple Tissues | No |
| 225 | 197 | 100 | **11** | / | IRL/907/2008 | 30/12/2007 | N752 | MA/NFD | PR77 | Multiple Tissues | No |
| 226 | 197 | 100 | **11** | / | IRL/585/2008 | 11/01/2008 | N752 | MA/NFD | PR77 | Multiple Tissues | No |
| 227 | 197 | 100 | **11** | / | IRL/849/2008 | 19/01/2008 | N752 | MA/NFD | PR77 | Multiple Tissues | No |
| 228 | 197 | 100 | **11** | / | IRL/470/2008 | 19/02/2008 | N752 | MA/NFD | PR77 | Multiple Tissues | No |
| 229 | 198 | 104 | **11** | 3 | IRL/471/2008 | 19/02/2008 | N752 | SA/NFD | PR55 | Multiple Tissues | No |
| 230 | 199 | 118 | **11** | 3 | IRL/966/2009 | 11/04/2009 | **D752** | MN | PR113 | Nasal swab | No |
| 231 | 199 | 118 | **11** | / | IRL/968/2009 | 12/04/2009 | **D752** | MN | PR113 | Multiple Tissues | No |
| 232 | 200 | 127 | **11** | 3 | IRL/307/2015 | 28/12/2015 | N752 | SA/NFD | PR49 | Multiple Tissues | No |
| 233 | 201 | 135 | **11** | / | IRL/082/2011 | 26/03/2011 | N752 | SA/NFD | PR69 | Multiple Tissues | No |
| 234 | 202 | 149 | **11** | 3 | IRL/684/2012 | 24/10/2012 | N752 | SA/NFD | PR109 | Multiple Tissues | No |
| 235 | 203 | 170 | **11** | 3 | IRL/705/2015 | 12/02/2015 | N752 | SA/NFD | PR34 | Multiple Tissues | No |
| 236 | 204 | 174 | **11** | 3 | IRL/286/2015 | 23/03/2015 | N752 | SA/NFD | PR66 | Multiple Tissues | No |
| 237 | 205 | 181 | **11** | 3 | IRL/776/2015 | 13/11/2015 | N752 | MA/NFD | PR36 | Multiple Tissues | No |
| 238 | 205 | 181 | **11** | / | IRL/035/2016 | 05/01/2016 | N752 | MA/NFD | PR36 | Multiple Tissues | No |
| 239 | 206 | 195 | **11** | 3 | IRL/160/2016 | 09/05/2016 | N752 | SA/NFD | PR47 | Multiple Tissues | No |
| 240 | 207 | 7 | **11** | Unassigned | IRL/618/1992 | 28/03/1992 | N752 | R | PR88 | Nasal swab | No |
| 241 | 208 | 11 | **11** | Unassigned | IRL/608/1993 | 02/04/1993 | N752 | SA/NFD | PR101 | Multiple Tissues | Yes, P1 |
| 242 | 209 | 14 | **11** | Unassigned | IRL/253/1994 | 19/02/1994 | N752 | SA/NFD | PR11 | Multiple Tissues | No |
| 243 | 210 | 30 | **11** | Unassigned | IRL/414/1998 | 03/04/1998 | N752 | SA/NFD | PR38 | Multiple Tissues | No |
| 244 | 211 | 35 | **11** | Unassigned | IRL/651/1999 | 17/04/1999 | N752 | SA/NFD | PR97 | Multiple Tissues | No |
| 245 | 212 | 41 | **11** | Unassigned | IRL/806/2001 | 22/03/2001 | N752 | SA/NFD | PR88 | Multiple Tissues | No |
| 246 | 213 | 44 | **11** | / | IRL/660/2001 | 20/04/2001 | N752 | SN | PR106 | Nasal swab | Yes, P1 |
| 247 | 214 | 47 | **11** | Unassigned | IRL/368/2002 | 04/04/2002 | N752 | SA/NFD | PR37 | Multiple Tissues | No |
| 248 | 215 | 54 | **11** | Unassigned | IRL/026/2004 | 24/05/2004 | N752 | SA/NFD | PR34 | Multiple Tissues | No |
| 249 | 216 | 57 | **11** | Unassigned | IRL/187/2005 | 01/02/2005 | N752 | SA/NFD | PR57 | Multiple Tissues | No |
| 250 | 217 | 63 | **11** | Unassigned | IRL/276/2005 | 22/03/2005 | N752 | MA/NFD | PR85 | Multiple Tissues | No |
| 251 | 218 | 98 | **11** | Unassigned | IRL/228/2007 | 15/04/2007 | N752 | SA/NFD | PR86 | Multiple Tissues | No |
| 252 | 219 | 102 | **11** | Unassigned | IRL/858/2008 | 07/02/2008 | N752 | SA/NFD | PR63 | Multiple Tissues | No |
| 253 | 220 | 110 | **11** | Unassigned | IRL/557/2008 | 30/04/2008 | N752 | SA/NFD | PR3 | Multiple Tissues | No |
| 254 | 221 | 126 | **11** | Unassigned | IRL/349/2010 | 21/03/2010 | N752 | SN | PR2 | Multiple Tissues | No |
| 255 | 222 | 152 | **11** | / | IRL/609/2013 | 25/02/2013 | N752 | SA/NFD | PR23 | Multiple Tissues | No |
| 256 | 223 | 154 | **11** | / | IRL/697/2013 | 05/04/2013 | N752 | SA/NFD | PR36 | Multiple Tissues | No |
| 257 | 224 | 211 | **11** | / | IRL/597/1998 | 16/04/1998 | N752 | SA/NFD | PR27 | Multiple Tissues | No |
|  |  |  |  |  |  |  |  |  |  |  |  |
| 258 | 225 | 23 | **13** | 2 | IRL/741/1996 | 07/02/1996 | N752 | SA/NFD | PR56 | Multiple Tissues | No |
| 259 | 226 | 62 | **13** | 2 | IRL/127/2005 | 21/03/2005 | N752 | MA/NFD | PR108 | Multiple Tissues | No |
| 260 | 226 | 62 | **13** | / | IRL/894/2005 | 25/03/2005 | N752 | MA/NFD | PR108 | Multiple Tissues | No |
| 261 | 227 | 70 | **13** | 2 | IRL/383/2005 | 31/03/2005 | N752 | SA/NFD | PR94 | Multiple Tissues | No |
| 262 | 228 | 108 | **13** | 2 | IRL/003/2008 | 09/04/2008 | N752 | SA/NFD | PR75 | Multiple Tissues | No |
| 263 | 229 | 137 | **13** | 2 | IRL/099/2011 | 21/04/2011 | N752 | SA/NFD | PR74 | Multiple Tissues | No |
| 264 | 230 | 141+T3 | **13** | 2 | IRL/350/2011 | 08/06/2011 | **D752** | MN | PR96 | Nasal swab | No |
| 265 | 231 | 142+T3 | **13** | 2 | IRL/331/2011 | 08/06/2011 | **D752** | MN | PR96 | Nasal swab | No |
| 266 | 232 | 157 | **13** | 2 | IRL/276/2013 | 19/04/2013 | N752 | SA/NFD | PR30 | Multiple Tissues | No |
| 267 | 233 | 168 | **13** | 2 | IRL/912/2014 | 03/12/2014 | N752 | MA/NFD | PR46 | Multiple Tissues | Yes, P1 |
| 268 | 234 | 187 | **13** | 2 | IRL/791/2016 | 05/03/2016 | N752 | SA/NFD | PR36 | Multiple Tissues | No |
| 269 | 235 | 188 | **13** | 2 | IRL/526/2016 | 25/03/2016 | N752 | SA/NFD | PR88 | Multiple Tissues | No |
| 270 | 236 | 217 | **13** | 2 | IRL/079/2006 | 29/04/2006 | N752 | SA/NFD | PR84 | Multiple Tissues | No |
| 271 | 237 | 218 | **13** | 2 | IRL/411/2006 | 04/05/2006 | N752 | MA/NFD | PR76 | Multiple Tissues | No |
| 272 | 238 | 219 | **13** | 2 | IRL/847/2006 | 28/02/2006 | N752 | MA/NFD | PR112 | Multiple Tissues | No |

^1^ The same outbreak is indicated by the same number and highlighted in blue.

^2^ Same premises are indicated by the same number and highlighted in red. +T: denotes linked premises. There are a total of five epidemiological links, T1-T5.

^3^ U_L_ clade number correlates to that described by Bryant *et al*. (2018).

^4^ ORF68 group correlates to that described by Nugent *et al*. (2006).

^5^ The putative neurological marker in the polymerase gene ORF30 at aa position 752 is highlighted in bold.

^6^ Multiple tissues from abortion/neonatal foal death includes lungs, liver, spleen, allantochorion.

^7^ One passage in cell culture.

^8^ Not applicable.

Abbreviations: IRL, Ireland; ITA, Italy.
