# Supplementary material for: Molecular Characterisation of Equine Herpesvirus 1 Isolates from Cases of Abortion, Respiratory and Neurological Disease in Ireland between 1990 and 2017
Source: Pathogens. 2019 Jan 15;8(1):7. doi: 10.3390/pathogens8010007 (PMC6471309; doi:10.3390/pathogens8010007)
Supplement: Supplementary file 1 [file pathogens-08-00007-s001.zip › SupplementaryData/Supplementary Table S2.docx]

Supplementary Table S2: Accession codes for EHV-1 sequences used in alignment.

Accession codes for complete and partial genome sequences used in alignment Supplementary Figure S1. Sequences included in phylogenetic tree (Figure 1) are highlighted in bold.

| **Genbank Accession** | **U_L_ clade** | **Country** | **Strain Name** |
| --- | --- | --- | --- |
| AB992258.1 | 1 | Japan | **EHV-1.HH1** |
| AY464052.1 | 9 | UK | **EHV-1.V592** |
| AY665713.1 | 1 | UK | **EHV-1.Ab4** |
| KF644566.1 | 3 | Japan | **EHV-1.90C16** |
| KF644567.1 | 7 | USA | **EHV-1.FL06** |
| KF644568.1 | 8 | UK | **EHV-1.NMKT04** |
| KF644569.1 | 2 | USA | **EHV-1.NY03** |
| KF644570.1 | 6 | USA | **EHV-1.NY05** |
| KF644571.1 | 5 | USA | EHV-1.OH03 |
| KF644572.1 | 6 | USA | EHV-1.VA02 |
| KF644576.1 | 3 | Japan | **EHV-1.00C19** |
| KF644577.1 | 3 | Japan | EHV-1.89C105 |
| KF644578.1 | 3 | Japan | **EHV-1.01C1** |
| KF644579.1 | 3 | Japan | **EHV-1.89C25** |
| KM593996.1 | 5 | USA | **EHV-1.T953** |
| KT324724.1 | 6 | New Zealand | EHV-1.NZA-77 |
| KT324725.1 | 3 | Australia | EHV-1.3045-07 |
| KT324726.1 | 7 | Australia | **EHV-1.3038-07** |
| KT324727.1 | 6 | Australia | **EHV-1.2222-03** |
| KT324728.1 | 7 | Australia | EHV-1.2019-02 |
| KT324729.1 | 7 | Australia | **EHV-1.196-02** |
| KT324730.1 | 1 | Australia | **EHV-1.1074-94** |
| KT324731.1 | 6 | Australia | EHV-1.1029-93 |
| KT324732.1 | 7 | Australia | EHV-1.970-90 |
| KT324733.1 | 3 | Australia | EHV-1.717A-82 |
| KT324734.1 | 7 | Australia | EHV-1.438-77 |
| KU206425 | 7 | UK | Suffolk/48/2013 |
| KU206426 | 7 | UK | Suffolk/125/2013 |
| KU206440 | 10 | UK | Devon/28/2003 |
| KU206441 | 10 | UK | Suffolk/82/2013 |
| KU206442 | 10 | UK | **Suffolk/89/2013** |
| KU206443 | 10 | UK | **Suffolk/87/2009** |
| KU206444 | 11 | UK | **UK/58/2003** |
| KU206445 | 11 | UK | **Gloucestershire/127/1998** |
| KU206446 | 11 | UK | Gloucestershire/77/2013 |
| KU206447 | 11 | UK | **Gloucestershire/54/2013** |
| KU206451 | 9 | UK | **Bristol/2/1993** |
| KU206454 | 8 | UK | **Oxfordshire/27/2011** |
| KU206462 | 1 | UK | Hampshire/1/2008 |
| KU206463 | 1 | UK | **Berkshire/7/1996** |
| KU206464 | 1 | UK  U | **Leicestershire/22/1996** |
| KU206465 | 1 | UK | **UK/32/1982** |
| KU206466 | 1 | UK | Hertfordshire/188/2010 |
| KU206467 | 1 | UK | **Hong Kong/57/1984** |
| KU206470 | 5 | UK | **Oxfordshire/206/2013** |
| KU206474 | 6 | UK | Suffolk/10/2012 |
| KU206475 | 6 | UK | **Lincolnshire/2/2012** |
| KU206479 | 12 | UK | Suffolk/91/1994 |
| KU206480 | 13 | UK | Suffolk/123/2005 |
