# Supplementary material for: Molecular Characterisation of Equine Herpesvirus 1 Isolates from Cases of Abortion, Respiratory and Neurological Disease in Ireland between 1990 and 2017
Source: Pathogens. 2019 Jan 15;8(1):7. doi: 10.3390/pathogens8010007 (PMC6471309; doi:10.3390/pathogens8010007)
Supplement: Supplementary file 1 [file pathogens-08-00007-s001.zip › SupplementaryData/Supplementary Table S4.docx]

Supplementary Table S4: Details of EHV-1 ORF68 sequences used in network analysis

| **Node** | **Country** | **GenBank Accession Code** | **Year** | **Reference** |
| --- | --- | --- | --- | --- |
| A | Japan | KF644566.1 | / | / |
| A | UK | DQ172353 | 1980 | [1] |
| A | UK | DQ172356 | 1983 | [1] |
| A | UK | DQ172358 | 1983 | [1] |
| A | UK | DQ172369 | 1993 | [1] |
| B | Argentina | DQ172311 | 1990 | [1] |
| B | Argentina | DQ172312 | 1990 | [1] |
| B | Argentina | DQ172313 | 1991 | [1] |
| B | Argentina | DQ172314 | 1991 | [1] |
| B | Argentina | DQ172315 | 1991 | [1] |
| B | Argentina | DQ172316 | 1996 | [1] |
| B | Argentina | DQ172308 | 2000 | [1] |
| B | Australia | DQ172318 | 1982 | [1] |
| B | Australia | KT324733 | 1982 | [2] |
| B | Australia | KT324730 | 1994 | [2] |
| B | Australia | KT324725 | 2007 | [2] |
| B | Belgium | GU271938 | 2009 | [3] |
| B | France | DQ172328 | 1997 | [1] |
| B | France | DQ172329 | 1999 | [1] |
| B | France | DQ172330 | 1999 | [1] |
| B | France | DQ172331 | 1999 | [1] |
| B | Hungary | HQ654053 | 1977 | [4] |
| B | Hungary | HQ654058 | 1982 | [4] |
| B | Hungary | HQ654061 | 1988 | [4] |
| B | Hungary | HQ654062 | 1988 | [4] |
| B | Hungary | HQ654063 | 1990 | [4] |
| B | Hungary | HQ654065 | 1991 | [4] |
| B | Hungary | HQ654066 | 1992 | [4] |
| B | Hungary | HQ654080 | 1997 | [4] |
| B | Hungary | HQ654076 | 1998 | [4] |
| B | Hungary | HQ654077 | 1998 | [4] |
| B | Hungary | HQ654078 | 1998 | [4] |
| B | Hungary | HQ654070 | 2003 | [4] |
| B | Hungary | HQ654072 | 2004 | [4] |
| B | Hungary | HQ654082 | 2005 | [4] |
| B | Japan | KF644576.1 | / | / |
| B | Japan | KF644577.1 | / | / |
| B | Japan | KF644579.1 | / | / |
| B | UK | DQ172352 | 1979 | [1] |
| B | UK | DQ172360 | 1986 | [1] |
| B | UK | DQ172370 | 1999 | [1] |
| B | UK | DQ172338 | 2003 | [1] |
| B | USA | DQ172390 | 1970 | [1] |
| B | USA | DQ172393 | 1976 | [1] |
| B | USA | DQ172397 | 1983 | [1] |
| B | USA | DQ172399 | 1984 | [1] |
| B | USA | DQ172401 | 1985 | [1] |
| B | USA | DQ172403 | 1985 | [1] |
| B | USA | DQ172405 | 1986 | [1] |
| B | USA | DQ172406 | 1986 | [1] |
| B | USA | DQ172409 | 1990 | [1] |
| B | USA | DQ172413 | 1999 | [1] |
| B | USA | DQ172374 | 2000 | [1] |
| B | USA | DQ172380 | 2003 | [1] |
| B | USA | DQ172381 | 2003 | [1] |
| B | USA | DQ172382 | 2003 | [1] |
| B | USA | DQ172383 | 2003 | [1] |
| B | USA | DQ172386 | 2003 | [1] |
| C | Australia | KT324734 | 1977 | [2] |
| C | Australia | KT324734 | 1977 | [2] |
| C | Australia | KT324732 | 1990 | [2] |
| C | Australia | DQ172317 | 2002 | [1] |
| C | Australia | KT324728 | 2002 | [2] |
| C | Australia | KT324729 | 2002 | [2] |
| C | Australia | KT324726 | 2007 | [2] |
| C | Belgium | DQ172320 | 1994 | [1] |
| C | Belgium | DQ172321 | 1995 | [1] |
| C | Belgium | DQ172322 | 1997 | [1] |
| C | Belgium | DQ172323 | 1999 | [1] |
| C | Belgium | DQ172324 | 1999 | [1] |
| C | Belgium | DQ172319 | 2003 | [1] |
| C | Belgium | GU271941 | 2003 | [3] |
| C | Belgium | GU271940 | 2009 | [3] |
| C | France | DQ172327 | 2002 | [1] |
| C | Hungary | HQ654081 | 1997 | [4] |
| C | Hungary | HQ654071 | 2003 | [4] |
| C | Hungary | HQ654068 | 2004 | [4] |
| C | Hungary | HQ654083 | 2006 | [4] |
| C | Hungary | HQ654087 | 2008 | [4] |
| C | Netherlands | DQ172371 | 1995 | [1] |
| C | Netherlands | DQ172372 | 1999 | [1] |
| C | Poland | KY201135.1 | 1999 | [5] |
| C | Poland | KY201140.1 | 2004 | [5] |
| C | Poland | KY201147.1 | 2008 | [5] |
| C | UK | DQ172357 | 1983 | [1] |
| C | UK | DQ172363 | 1988 | [1] |
| C | UK | DQ172367 | 1991 | [1] |
| C | UK | DQ172368 | 1993 | [1] |
| C | UK | DQ172333 | 2000 | [1] |
| C | UK | DQ172334 | 2001 | [1] |
| C | UK | DQ172336 | 2002 | [1] |
| C | UK | DQ172337 | 2003 | [1] |
| C | UK | DQ172339 | 2003 | [1] |
| C | UK | DQ172340 | 2003 | [1] |
| C | UK | DQ172341 | 2003 | [1] |
| C | UK | DQ172342 | 2004 | [1] |
| C | UK | DQ172343 | 2004 | [1] |
| C | UK | DQ172346 | 2004 | [1] |
| C | UK | DQ172348 | 2004 | [1] |
| C | UK | DQ172349 | 2004 | [1] |
| C | UK | DQ172350 | 2004 | [1] |
| C | UK | DQ172351 | 2005 | [1] |
| C | USA | DQ172404 | 1986 | [1] |
| C | USA | DQ172410 | 1990 | [1] |
| C | USA | DQ172411 | 1990 | [1] |
| C | USA | DQ172412 | 1992 | [1] |
| C | USA | KF644567.1 | 2006 | / |
| D | Belgium | GU271939 | 2009 | [3] |
| D | Ethiopia | KP765722 | 2013 | [6] |
| D | Ethiopia | KP765723 | 2013 | [6] |
| D | Ethiopia | KP765724 | 2013 | [6] |
| D | France | DQ172326 | 2000 | [1] |
| D | Germany | KJ513013 | 2012 | [7] |
| D | Hungary | HQ654064 | 1990 | [4] |
| D | Hungary | HQ654085 | 2007 | [4] |
| D | Hungary | HQ654086 | 2007 | [4] |
| D | India | KT180205 | 1990 | [8] |
| D | India | KM285388 | 1998 | [8] |
| D | India | KM285390 | 2013 | [8] |
| D | India | KM285391 | 2014 | [8] |
| D | India | KT180209 | 2014 | [8] |
| D | India | KT180210 | 2014 | [8] |
| D | India | KT180211 | 2014 | [8] |
| D | Poland | KY201143.1 | 2005 | [5] |
| D | Poland | KY201149.1 | 2009 | [5] |
| D | Poland | KY201161.1 | 2013 | [5] |
| D | Poland | KY201162.1 | 2013 | [5] |
| D | UK | DQ172354 | 1981 | [1] |
| D | UK | DQ172355 | 1981 | [1] |
| D | UK | DQ172344 | 2004 | [1] |
| D | UK | DQ172345 | 2004 | [1] |
| D | UK | DQ172347 | 2004 | [1] |
| D | USA | DQ172407 | 1986 | [1] |
| E | Australia | KT324727 | 2003 | [2] |
| E | Canada | DQ172325 | 1989 | [1] |
| E | India | KM285386 | 1996 | [8] |
| E | India | KT180206 | 1996 | [8] |
| E | India | KT180207 | 1997 | [8] |
| E | India | KM285387 | 1998 | [8] |
| E | India | KM285389 | 2007 | [8] |
| E | New Zealand | KT324724.1 | / | [2] |
| E | Poland | DQ172373 | 1968 | [1] |
| E | USA | DQ172389 | 1941 | [1] |
| E | USA | DQ172391 | 1972 | [1] |
| E | USA | DQ172392 | 1975 | [1] |
| E | USA | DQ172395 | 1981 | [1] |
| E | USA | DQ172396 | 1981 | [1] |
| E | USA | DQ172398 | 1983 | [1] |
| E | USA | DQ172402 | 1985 | [1] |
| E | USA | DQ172414 | 1999 | [1] |
| E | USA | DQ172415 | 1999 | [1] |
| E | USA | DQ172375 | 2001 | [1] |
| E | USA | DQ172376 | 2001 | [1] |
| E | USA | DQ172377 | 2002 | [1] |
| E | USA | DQ172378 | 2003 | [1] |
| E | USA | DQ172379 | 2003 | [1] |
| E | USA | DQ172387 | 2003 | [1] |
| E | USA | KF644570.1 | 2005 | / |
| F | Poland | KY201136.1 | 1999 | [5] |
| F | Poland | KY201138.1 | 2002 | [5] |
| F | Poland | KY201139.1 | 2003 | [5] |
| F | Poland | KY201141.1 | 2004 | [5] |
| F | Poland | KY201142.1 | 2004 | [5] |
| F | Poland | KY201144.1 | 2006 | [5] |
| F | Poland | KY201146.1 | 2007 | [5] |
| F | Poland | KY201148.1 | 2009 | [5] |
| F | Poland | KY201150.1 | 2010 | [5] |
| F | Poland | KY201151.1 | 2010 | [5] |
| F | Poland | KY201153.1 | 2011 | [5] |
| F | Poland | KY201154.1 | 2012 | [5] |
| F | Poland | KY201156.1 | 2012 | [5] |
| F | Poland | KY201157.1 | 2012 | [5] |
| F | Poland | KY201158.1 | 2012 | [5] |
| F | Poland | KY201159.1 | 2012 | [5] |
| F | Poland | KY201160.1 | 2013 | [5] |
| F | Poland | KY201167.1 | 2014 | [5] |
| F | Poland | KY201168.1 | 2014 | [5] |
| F | Poland | KY201170.1 | 2015 | [5] |
| F | Poland | KY201171.1 | 2015 | [5] |
| F | UK | DQ172361 | 1986 | [1] |
| G | Hungary | HQ654075 | 1997 | [4] |
| G | Hungary | HQ654074 | 1998 | [4] |
| G | Hungary | HQ654079 | 1998 | [4] |
| G | Hungary | HQ654067 | 2004 | [4] |
| G | Hungary | HQ654084 | 2006 | [4] |
| H | Hungary | HQ654054 | 1978 | [4] |
| H | Hungary | HQ654055 | 1978 | [4] |
| H | Hungary | HQ654056 | 1978 | [4] |
| H | Hungary | HQ654057 | 1982 | [4] |
| H | Hungary | HQ654059 | 1982 | [4] |
| I | Poland | KY201163.1 | 2013 | [5] |
| I | Poland | KY201164.1 | 2013 | [5] |
| I | Poland | KY201165.1 | 2013 | [5] |
| I | Poland | KY201166.1 | 2013 | [5] |
| I | Poland | KY201172.1 | 2015 | [5] |
| J | Poland | KY201137.1 | 2001 | [5] |
| J | Poland | KY201145.1 | 2006 | [5] |
| J | Poland | KY201152.1 | 2010 | [5] |
| J | Poland | KY201155.1 | 2012 | [5] |
| K | UK | DQ172359 | 1985 | [1] |
| K | UK | DQ172366 | 1991 | [1] |
| K | UK | DQ172335 | 2001 | [1] |
| L | UK | DQ172364 | 1989 | [1] |
| L | UK | DQ172365 | 1989 | [1] |
| M | USA | DQ172385 | 2003 | [1] |
| M | USA | DQ172388 | 2003 | [1] |
| N | Australia | KF434389 | 1993 | [9] |
| N | Australia | KT324731 | 1993 | [2] |
| O | Hungary | HQ654069 | 2004 | [4] |
| P | Hungary | HQ654060 | 1983 | [4] |
| Q | Argentina | DQ172309 | 1979 | [1] |
| R | USA | DQ172408 | 1989 | [1] |
| S | Australia | KF434390 | 1999 | [9] |
| T | USA | DQ172394 | 1979 | [1] |
| U | UK | DQ172362 | 1987 | [1] |
| V | USA | DQ172384 | 2003 | [1] |
| W | UK | DQ172332 | 2000 | [1] |
| X | Hungary | HQ654073 | 2001 | [4] |
| Y | India | KM285392 | 1990 | [8] |

**References**

1. Nugent, J.; Birch-Machin, I.; Smith, K.C.; Mumford, J.A.; Swann, Z.; Newton, J.R.; Bowden, R.J.; Allen, G.P.; Davis-Poynter, N. Analysis of equid herpesvirus 1 strain variation reveals a point mutation of the DNA polymerase strongly associated with neuropathogenic versus nonneuropathogenic disease outbreaks. *J Virol* **2006**, *80*, 4047-4060, doi:10.1128/jvi.80.8.4047-4060.2006.

2. Vaz, P.K.; Horsington, J.; Hartley, C.A.; Browning, G.F.; Ficorilli, N.P.; Studdert, M.J.; Gilkerson, J.R.; Devlin, J.M. Evidence of widespread natural recombination among field isolates of equine herpesvirus 4 but not among field isolates of equine herpesvirus 1. *The Journal of general virology* **2016**, *97*, 747-755, doi:10.1099/jgv.0.000378.

3. Gryspeerdt, A., Vanderkerckhove, A., Van Doorsselaere, J., Van de Walle, G., & Nauwynck, H. . Description of an unusually large outbreak of nervous system disorders caused by equine herpesvirus 1 (EHV1) in 2009 in Belgium. *Vlaams Diergeneeskundig Tijdschrif* **2011**, *80*, 147-153.

4. Malik, P.; Balint, A.; Dan, A.; Palfi, V. Molecular characterisation of the ORF68 region of equine herpesvirus-1 strains isolated from aborted fetuses in Hungary between 1977 and 2008. *Acta veterinaria Hungarica* **2012**, *60*, 175-187, doi:10.1556/AVet.2012.015.

5. Stasiak, K.; Dunowska, M.; Hills, S.F.; Rola, J. Genetic characterization of equid herpesvirus type 1 from cases of abortion in Poland. *Archives of virology* **2017**, 10.1007/s00705-017-3376-3, 2329–2233, doi:10.1007/s00705-017-3376-3.

6. Negussie, H.; Gizaw, D.; Tessema, T.S.; Nauwynck, H.J. Equine Herpesvirus-1 Myeloencephalopathy, an Emerging Threat of Working Equids in Ethiopia. *Transboundary and emerging diseases* **2017**, *64*, 389-397, doi:10.1111/tbed.12377.

7. Damiani, A.M.; de Vries, M.; Reimers, G.; Winkler, S.; Osterrieder, N. A severe equine herpesvirus type 1 (EHV-1) abortion outbreak caused by a neuropathogenic strain at a breeding farm in northern Germany. *Veterinary microbiology* **2014**, *172*, 555-562, doi:<http://dx.doi.org/10.1016/j.vetmic.2014.06.023>.

8. Anagha, G.; Gulati, B.R.; Riyesh, T.; Virmani, N. Genetic characterization of equine herpesvirus 1 isolates from abortion outbreaks in India. *Archives of virology* **2017**, *162*, 157-163, doi:10.1007/s00705-016-3097-z.

9. Cuxson, J.L.; Hartley, C.A.; Ficorilli, N.P.; Symes, S.J.; Devlin, J.M.; Gilkerson, J.R. Comparing the genetic diversity of ORF30 of Australian isolates of 3 equid alphaherpesviruses. *Veterinary microbiology* **2014**, *169*, 50-57, doi:<http://dx.doi.org/10.1016/j.vetmic.2013.12.007>.
