# Supplementary material for: Molecular Characterisation of Equine Herpesvirus 1 Isolates from Cases of Abortion, Respiratory and Neurological Disease in Ireland between 1990 and 2017
Source: Pathogens. 2019 Jan 15;8(1):7. doi: 10.3390/pathogens8010007 (PMC6471309; doi:10.3390/pathogens8010007)
Supplement: Supplementary file 1 [file pathogens-08-00007-s001.zip › SupplementaryData/Supplementary Table S5.docx]

Supplementary Table S5. Primer sequences used for multi-locus amplification

| **Primer name^1^** | **Primer Sequence 5` to 3`** | **Position on Ab4 strain** | **Amplicon Size** |
| --- | --- | --- | --- |
| ORF 2.F | TGTAGTAGTGGGTGAGTCGGTAAG | 2242-2265 | 291bp |
| ORF 2.R | GTCTCAAACCCTACCACAATCAAC | 2532-2509 |  |
| ORF 5.F | TGTATTAGAGCCGTGGTTAGC | 5293-5313 | 522bp |
| ORF 5.R | TCTGACACCGAAGACAACTTCA | 5814-5793 |  |
| ORF 8.F | AAGTGGCTGATGTCAAGTCTATGT | 10306-10329 | 568bp |
| ORF 8.R | ATCTCGGCTACGGCATTCAC | 10874-10855 |  |
| ORF 11.F | GTAAACTCCATCAAGAGCGGTAAG | 13050-13073 | 353bp |
| ORF 11.R | CGTCTAGAGGCTGTACGAGGTC | 13402-13381 |  |
| ORF 13.F | CAGCTTGTACGATAGCCAGTTT | 16060-16081 | 842bp |
| ORF 13.R | TGTCAAATGTACGCTGCTCAAG | 16901-16880 |  |
| ORF 14.F | CTGCGAGACTCCGATTACACT | 19895-19915 | 339bp |
| ORF 14.R | CTCTGCAGATTCCTCGTCAC | 20233-20214 |  |
| ORF 15.F | GTATGATGTGGTCCAGACGAAG | 20501-20522 | 270bp |
| ORF 15.R | CAGTATGCGAAGAGTACTCAGAGA | 20770-20747 |  |
| ORF 22.F | TCTACGTAACCTACCACGTAGACTG | 31387-31411 | 403bp |
| ORF 22.R | GTCAAGCAGTCGGTGTGAGC | 31789-31770 |  |
| ORF 29.F | CATATCTCTCGAACACACGCTCT | 50589-50611 | 384bp |
| ORF 29.R | ATAGCGAGAGACAGTTGTCAGCA | 50972-50950 |  |
| ORF 30.752.F | GTAGATAACCCTGACGGAGTA | 52530-52550 | 515bp |
| ORF 30.752.R | TGGTTGTGTTTGACTTCGCTA | 53044-53024 |  |
| ORF 30.990.F | AGCTCAGCAGTCATAACGAACTC | 52072-52094 | 207bp |
| ORF 30.990.R | GTACGCGCGACATCTGGTAG | 52278-52259 |  |
| ORF 31.F | CGGACTTACCGTCGAACAGA | 55602-55621 | 362bp |
| ORF 31.R | TGTTGCACACGTACACCATCT | 55963-55943 |  |
| ORF 32.F | GCCTCTATCAGCTATGGAGCAA | 59230-59251 | 392bp |
| ORF 32.R | GGTATTCGCACGGTTCGTCT | 59621-59602 |  |
| ORF 33.15.F | CTAGCCGCCGCAGTATTCTC | 61301-61320 | 237bp |
| ORF 33.15.R | GAGCACTGCATACAGGAGAGAG | 61537-61516 |  |
| ORF 33.976.F | TGATCGCCAGTAACGTCTCA | 64274-64293 | 208bp |
| ORF 33.976.R | TCGGCGGAGATACTAGGC | 64481-64464 |  |
| ORF 34.F | ACGCGGTATCTCCACAGCTA | 64589-64608 | 283bp |
| ORF 34.R | GTTGTCGTCTCGACAGCAGA | 64871-64852 |  |
| ORF 36.F | ACTGAGCGATTGTCCGATGTT | 68562- 68582 | 358bp |
| ORF 36.R | GTCCACTATACCGACAGACGTG | 68919-68898 |  |
| ORF 37.F | ATCTCCACCGCGACTATAAGGT | 68974-68995 | 280bp |
| ORF 37.R | GGAGCCAGTGCCATCTATAAC | 69253-69233 |  |
| ORF 39.F | CATGGACCAGTTTGACGTTG | 72463-72482 | 238bp |
| ORF 39.R | TAGCACCTCGCGCATTGT | 72700-72683 |  |
| ORF 40.F | TAGTTGTAGTGATTGTAGCGATGC | 75391-75414 | 447bp |
| ORF 40.R | GTAACGTCTGGACTCATCGTATCT | 75837-75814 |  |
| ORF 42.F | AACTCTTACGGCGATAGATTGTA | 81450-81472 | 261bp |
| ORF 42.R | GCTGAGTAGAGGATACGCTTCTTG | 81710-81687 |  |
| ORF 45.F | CTACTCCGGTCGTTGAGTCC | 44050-44069 | 588bp |
| ORF 45.R | GATGTTGAATTGCAGCAGTAGG | 44637-44616 |  |
| ORF 46.F | AGTTTCACATGCTGGTAACCTTC | 86918-86940 | 254bp |
| ORF 46.R | CAGGACGCAACAGTCGTACTT | 87171-87151 |  |
| ORF 50.F | ACTCGACTCAGACTACATTGGA | 92054-92075 | 367bp |
| ORF 50.R | GGAGTAATAGTCTCCGACTCAAGG | 92420-92397 |  |
| ORF 52.F | CACTGGCACGTTGATGCTAT | 93104-93123 | 312bp |
| ORF 52.R | TAGTCGTGAGACTGGTCAGAGCTA | 93415-93392 |  |
| ORF 57.F | AACATGCCTCGCATGGTTCT | 104649-104668 | 369bp |
| ORF 57.R | ATACACGATCTGGACGTCTCTGT | 105017-104995 |  |
| ORF 67.F | GCTGAAACGCTCAATACCAC | 124310-124329 | 236bp |
| ORF 67.R | TCGAGTACCAGTTGCACGTC | 124545-124526 |  |
| ORF 73.F | AACTACTCCAGCGTTTGCTACACT | 133130-133151 | 338bp |
| ORF 73.R | GACACATAGCTCGTTGTAGGTTCT | 133467-133444 |  |
| ORF 76.F | ACTACACGTACACCGCTGCTT | 136976-136996 | 400bp |
| ORF 76.R | ATCACGCCTGCGACAGTAA | 137375-137357 |  |

^1^Forward and reverse primers are labelled according to the open reading frame targeted. Two ORFs (30 and 33) required two primer sets and are labelled according to the amino acid change targeted.
